# Dissecting the Interplay Mechanism among Process Parameters toward the Biofabrication of High-Quality Shapes in Embedded Bioprinting

**DOI:** 10.1002/adfm.202313088

**Published:** 2024-01-30

**Authors:** Yang Wu, Xue Yang, Deepak Gupta, Mecit Altan Alioglu, Minghao Qin, Veli Ozbolat, Yao Li, Ibrahim T. Ozbolat

**Affiliations:** School of Mechanical Engineering and Automation, Harbin Institute of Technology, Shenzhen 518055, China; School of Mechanical Engineering and Automation, Harbin Institute of Technology, Shenzhen 518055, China; The Huck Institutes of the Life Sciences, Penn State University University Park, PA 16802, USA; Engineering Science and Mechanics Department, Penn State University, University Park, PA 16802, USA; The Huck Institutes of the Life Sciences, Penn State University University Park, PA 16802, USA; Engineering Science and Mechanics Department, Penn State University, University Park, PA 16802, USA; School of Mechanical Engineering and Automation, Harbin Institute of Technology, Shenzhen 518055, China; Biotechnology Research and Application Center, Cukurova University, Adana 01130, Turkey; Ceyhan Engineering Faculty, Mechanical Engineering Department, Cukurova University, Adana 01330, Turkey; Institute of Natural and Applied Sciences, Tissue Engineering Department, Cukurova University, Adana 01130, Turkey; School of Mechanical Engineering and Automation, Harbin Institute of Technology, Shenzhen 518055, China; The Huck Institutes of the Life Sciences, Penn State University University Park, PA 16802, USA; Engineering Science and Mechanics Department, Penn State University, University Park, PA 16802, USA; Department of Biomedical Engineering, Penn State University, University Park, PA 16802, USA; Materials Research Institute, Penn State University, University Park, PA 16802, USA; Department of Neurosurgery, Penn State College of Medicine, Hershey, PA 17033, USA; Penn State Cancer Institute, Penn State University, Hershey, PA 17033, USA

**Keywords:** embedded bioprinting, print quality, print stability, resolution, support bath

## Abstract

Embedded bioprinting overcomes the barriers associated with the conventional extrusion-based bioprinting process as it enables the direct deposition of bioinks in 3D inside a support bath by providing in situ self-support for deposited bioinks during bioprinting to prevent their collapse and deformation. Embedded bioprinting improves the shape quality of bioprinted constructs made up of soft materials and low-viscosity bioinks, leading to a promising strategy for better anatomical mimicry of tissues or organs. Herein, the interplay mechanism among the printing process parameters toward improved shape quality is critically reviewed. The impact of material properties of the support bath and bioink, printing conditions, cross–linking mechanisms, and post-printing treatment methods, on the printing fidelity, stability, and resolution of the structures is meticulously dissected and thoroughly discussed. Further, the potential scope and applications of this technology in the fields of bioprinting and regenerative medicine are presented. Finally, outstanding challenges and opportunities of embedded bioprinting as well as its promise for fabricating functional solid organs in the future are discussed.

## 1. Introduction

In the past decades, 3D bioprinting has shown exceptional potential and played an increasingly important role in tissue engineering and organ manufacturing. Currently, there are four major 3D bioprinting modalities, namely extrusion-based bioprinting (EBB),^[1–4]^ droplet-based bioprinting (DBB),^[5–7]^ laser-assisted bioprinting (LAB),^[8–10]^ and light-based bioprinting (LBB).^[11]^ Among them, the most commonly used modality is the EBB,^[12]^ in which a print nozzle extrudes a bioink and deposits continuous fibers along a planned path to achieve 3D structures.^[13,14]^ EBB has been widely explored in the field of tissue engineering and regenerative medicine to fabricate various functional structures with complex geometries because of its advantages such as high cost-effectiveness, ease of operation, and a wide range of material compatibility.^[15]^ However, conventional EBB strategies face several challenges during bioprinting. For example, EBB is tough to deal with fabricating constructs with overhangs, resulting in significant limitations in mimicking natural tissues. In addition, the liquid-to-solid conversion of the bioink during bioprinting needs to be fast and timely to prevent the bioinks from collapsing.^[16]^

In recent years, the strategy of 3D bioprinting in a support bath has gained intensive attention, and its unique bioprinting characteristics compensate for the limitations of conventional EBB. The emergence of embedded bioprinting, an innovative derivative of the conventional EBB method, has been making a new era in the field of bioprinting by bringing a support bath to the bioprinting process to allow freeform 3D movement of the printhead in the support bath.^[17]^ Embedded bioprinting provides in situ support for the bioink with the help of the support bath, which has a self-healing capacity allowing the transformation from a solid-like to a liquid state by moving the nozzle to permit the extrusion of the bioink, and further transforms from a fluidized state back to a solid-like state after the bioink deposition, thereby enclosing the bioink and maintaining the structural shape.^[17]^ As a result, the requirement for rapid solidification and cross–linking of the bioink is mitigated, thus increasing the acceptable range of bioinks. In addition, the support bath allows omnidirectional printing, which is a significant step ahead of the conventional EBB. For complex 3D structures with overhangs and hollow features, the support bath enables in situ support without the need for additional support structures, thus improving the bioprinting efficiency and print quality.

Over the past few years, embedded bioprinting has been successfully used to fabricate complex biological structures, such as heart models^[18–20]^ and vascularized tissues.^[21–23]^ Researchers have proposed several strategies with outstanding bioprinting capabilities, such as freeform reversible embedding of suspended hydrogels (FRESH),^[24,25]^ sacrificial writing into functional tissue (SWIFT),^[23]^ and so on. For example, Lee et al.^[18]^ present the FRESH v2.0 that successfully facilitated the extrusion of collagen fibers at a resolution (20 μm) by one order of magnitude better than FRESH v1.0 and also demonstrated the fabrication of human heart components from capillaries to the entire organ. In embedded bioprinting, shape fidelity can be broadly considered as the degree to which bioprinted features match the intended design. The popular way to quantify the fidelity is to analyze the cross–sectional size and shape of bioprinted fibers. For example, Ning et al.^[26]^ quantified bioprinting fidelity through printed strand diameter (Dr), uniformity (Ur), angle (*α*_*r*_), and area (A_r_). Others^[27]^ compared the diameter and geometric shape of bioprinted fibers with the theoretical needle size and categorized them into well-defined and irregular fibers to define and discuss the bioprinting fidelity. These strategies aim to improve the performance and extend the applications to fabricate well-defined complex 3D structures, which can satisfy the requirements of prototypes as well as specific bionic and functional properties. Therefore, the current article focuses on the bioprinting resolution and shape fidelity to better understand the embedded bioprinting process and the parameters affecting it qualitatively and quantitatively.

**Figure 1.**
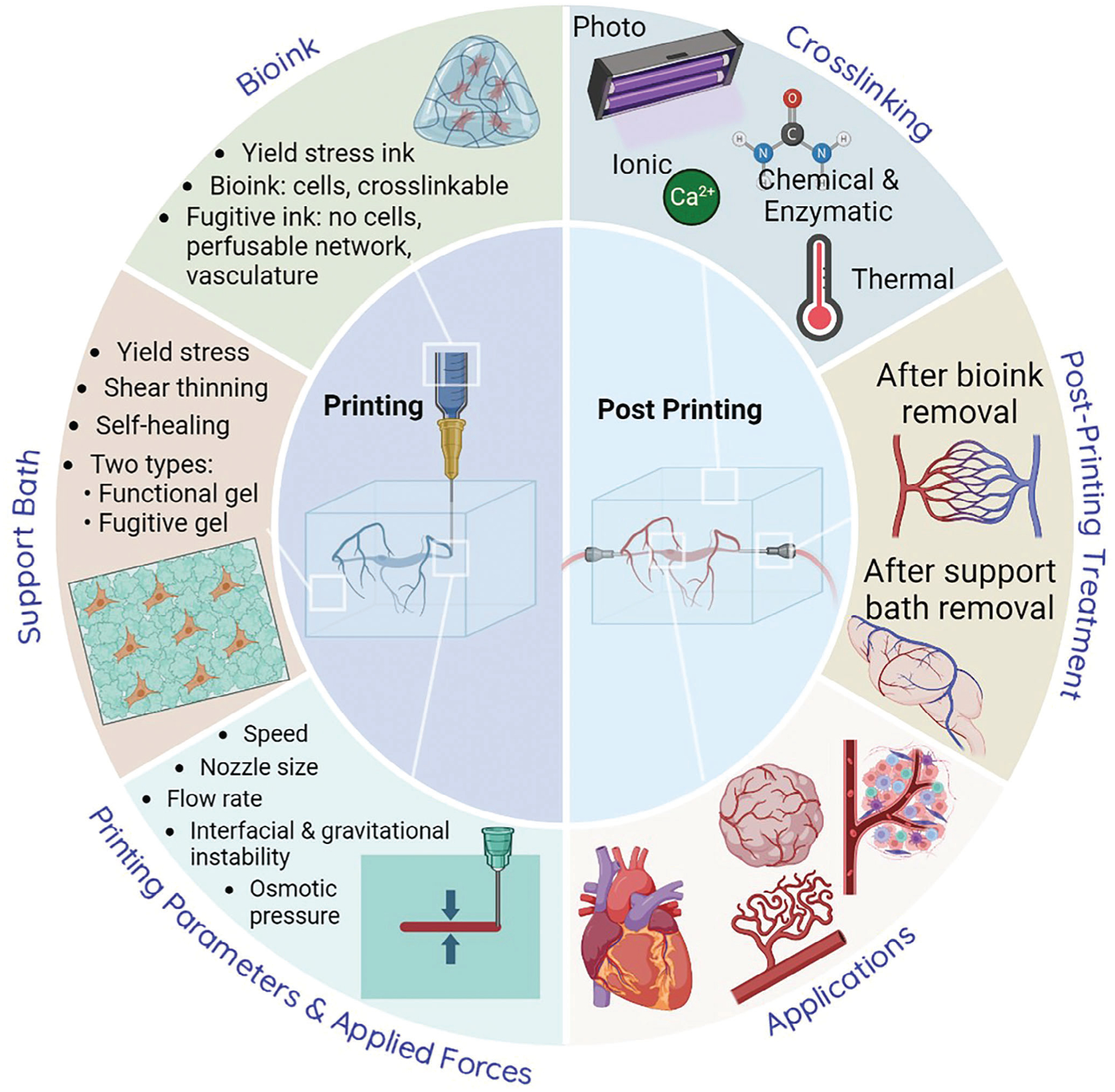
Illustration of all aspects associated with embedded printing, including bioinks, support baths, printing parameters, cross–linking techniques, post-printing treatments, and target applications.

Different embedded bioprinting strategies yield different bioprinting resolutions and fidelity, further determining the quality of bioprinted structures. Until now, review articles have focused on the ability of embedded bioprinting to create functional tissues and organs by discussing the material systems, basic theoretical strategies, and typical applications involved in embedded bioprinting.^[16,17,28–31]^ However, the impact of bioprinting strategies and conditions on the resolution, fidelity, and, thus, the shape quality of complex bioprinted structures has not been comprehensively and systematically reviewed. Therefore, in this article, we take the shape quality as the guide and explore the associated factors that govern it throughout the bioprinting process. These crucial factors, namely biomaterials for bioinks and support baths, bioprinting parameters, cross–linking mechanisms, and post-bioprinting treatments (see Figure 1), are systematically discussed, and their scope and limitations are expounded. First, the selection of biomaterials and the influence of their properties on shape quality is presented, followed by the discussion of the impact of bioprinting software and hardware settings on bioprinting stability, fidelity, and resolution. In addition, representative applications are demonstrated, and the effects of cross–linking mechanisms and post-processing strategies on shape quality were enlightened. Finally, current challenges experienced during embedded bioprinting are discussed, providing insights and perspectives to improve the success rate of bioprinting, the fidelity of bioprinted fibers, and the accuracy and stability of bioprinted constructs to benefit the future applications of orderly organization of functional tissues and organs.

## 2. Material Systems

To 3D bioprint complex-shaped tissues and organs, it is essential to select the proper materials to improve the bioprinting resolution and shape fidelity. This section discusses different material systems utilized in embedded bioprinting, including support baths and bioinks, and assesses critical properties suitable for such purposes.

### 2.1. Support Baths

#### 2.1.1. Evaluation of Support Bath Properties

The support bath, a crucial element of embedded bioprinting, offers a self-supporting environment for a bioink to prevent its instability and collapse after bioprinting, which is advantageous over the conventional EBB, where bioinks are deposited on a platform in the air. The support bath allows the deposition of bioinks in arbitrary positions and orientations. Thus, selecting the correct support bath material is one of the primary factors to ensure shape quality. The ideal support bath must have controlled rheological properties and be easily removed, facilitating the bioprinting and extraction of complex 3D structures.^[32]^ Rheological properties of the support bath are usually evaluated with a rheometer by conducting rotational, oscillation, and thixotropy testing.^[33–35]^ These materials often exhibit shear-thinning properties, viscoelasticity, and self-healing capability, which are further adjusted to achieve appealing bioprintability during extrusion.^[16,29,33,34]^

During the movement of the nozzle, the shear-thinning behavior of the support bath ensures that the shear stress generated by the nozzle surpasses the yield-stress of the support bath material, turning it into liquid locally around the nozzle and generating a smooth motion of the nozzle and continuous flow of the bioink in the support bath. Once the nozzle has passed through the support bath, the disturbed region of the support bath is required to self-heal. It should spontaneously fill up the resultant cavity created due to the nozzle movement and quickly return to the original solid-like state. In this manner, it confines and holds the bioprinted material stable at its location. The liquid-to-solid transition of the support bath enables the bioink to be smoothly deposited and firmly remain in the designated position in order to ensure the final shape of the structure. The self-healing property is vital for the shape quality of the final design and is generally estimated by the thixotropy test. The self-healing time of the support bath material refers to the time it takes to complete the liquid-solid phase transition (also called “thixotropy time”), which is derived from a thixotropy curve obtained by a three-stage test (i.e., low shear rate, high shear rate, and same low shear rate as in the first interval).^[33]^ The viscosity of the support bath decreases at high shear rates, and when the shear rate is reduced, the support bath immediately returns to 80 or 90% of the initial viscosity.^[32]^ Excessive thixotropic time should be avoided, preferably less than 1 s, to ensure that bioprinted fibers remain in place.^[16]^

The viscoelasticity of a support bath is usually characterized by oscillatory tests using a rheometer and the support bath exhibits viscous or elastic behavior over time. There are two types of oscillation tests, namely, amplitude and frequency sweep. The amplitude sweep is performed at a variable shear strain (e.g., 0.1%−100% strain) and a constant frequency (1 Hz) ,^[36]^ while the frequency sweep is performed at a variable frequency (e.g., 0.1–10 Hz) and a constant oscillation strain (typically within the linear viscoelastic region).^[37]^ The oscillation test yields a stress curve demonstrating the change of storage (*G*′) and loss modulus (*G*″) with strain. The stress value at the limit of the linear viscoelastic region (LVE) gives the yield-stress, which means the material starts to soften, while the intersection of *G*′ and *G*″ stress curves determines the flow stress value, which indicates that the support bath has a phase transition point (i.e., a change from solid-like (elastic) to liquid-like (viscous) behavior).^[33,38]^ When *G*′ > *G*″, the support bath has a solid-like behavior, while for *G2* < *G*″, the support bath exhibits a fluid-like behavior. The yield-stress and storage modulus of the support bath can be controlled by adjusting its concentration to ensure that the support bath provides the required support to extruded bioinks.^[39]^

In addition to the abovementioned properties, the support bath must be inert against the physical disturbances and chemical interactions of the environment, such as the change of magnetic/electric field and potential chemical reactions with cross–linking conditions. For temperature-sensitive materials, temperature variations lead to phase changes. Therefore, bioprinting and cross–linking temperatures must be accurately controlled for temperature-sensitive materials, such as gelatin and Pluronic.^[32,36,40]^ Also, it must provide a biocompatible environment for cells since cells may be in direct contact with the support bath.

#### 2.1.2. Support Bath Materials

Different support bath materials affect the fidelity and resolution of bioprinted structures differently. Two common types of support bath materials are discussed below, namely particle-based and continuous-phase support materials.

Hydrogel particles are typically compacted into a jammed state by centrifugation to develop particle-based support baths. During bioprinting, shear stress generated by the nozzle motion fluidizes the surrounding particles. As soon as the shear stress is released, the particles quickly return to their jammed state to hold the deposited bioink in place. It has been shown that particle-based support bath materials enable reversible solid-liquid phase transitions that are smooth and fast enough for high-fidelity purposes.^[41]^ Commonly reported materials that form hydrogel particles include alginate,^[42–44]^ agarose,^[45–47]^ Carbopol,^[48–50]^ gelatin,^[18,51,52]^ and their composites. Support bath materials made from hydrogel particles and the respective bioink used for embedded bioprinting are listed in Table 1.

Different methods of particle preparation affect the particle size, morphology, and size distribution, which in turn impact the rheological properties of the support bath (e.g., yield-stress, thixotropic behavior),^[53]^ and subsequently affect the fidelity and resolution of bioprinted structures. Kaitez et al.^[41]^ investigated the effect of different particle preparation methods on the size and yield-stress of alginate particles, including in-air microfluidics, microfluidic droplet formation, batch emulsion, and mechanical fragmentation. The reader is referred to the literature^[16,33]^ for more detailed information on methods pertaining the particle-based support preparation. In general, mechanical stirring allows for the generation of a large number of particles conveniently and the particle size can be controlled by varying the stirring time and rate.^[45]^ Fine and uniform-sized particles facilitate superior fidelity and resolution, while coarse and uneven particles lead to distortion of the fiber morphology and deteriorate the resolution.^[18]^ The size distribution of particles has notable effects on the fiber morphology. For example, a support bath consisting of low concentration and non-homogeneous large particles in jammed state results in larger gaps between particles, which causes the deposited bioink to diffuse through these gaps, thus increasing the diameter of the printed fibers and surface roughness.^[54]^ As the particle concentration increases and particle size decreases, these gaps get occupied, leading to smoother fibers with uniform diameters.^[32]^ Therefore, to facilitate high resolution and fidelity of the bioprinted structures within a particle-based support bath, especially for bioprinting of bioinks with low viscosity,^[32]^ a support bath composed of small-sized and highly concentrated particles is encouraged. Certainly, further research on process development to obtain particles with ease of handling, precise size, and uniform size distribution is expected.^[18,28,34,54]^ For example, FRESH v2.0 containing gelatin particles with uniform spherical morphology and particle diameter of 25 μm was prepared via a composite coalescence method.^[18]^ Bioprinted fibers had better shape fidelity, less roughness, and increased resolution by an order of magnitude as compared to FRESH v1.0.^[18]^ In addition, a uniform gellan gum bath with small and well-dispersed microgels has been generated based on the Hofmeister effect.^[55]^ By simply modifying the bath with the trisodium citrate (TSC), the average particle size of the bath with 0.6 mol L^−1^ TSC (30 ± 15 μm) was much smaller than that without adding TSC (350 ± 120 μm). The GG-TSC bath was capable of printing collagen fiber with a minimum diameter as thin as 25 μm, which led to a higher printing resolution than the GG-only bath. In another study, ion-modified polyvinyl alcohol (PVA) was applied to reduce the particle size.^[56]^ The average diameters of cationic PVA microgels were 18.5 ± 8.7 μm, which were much smaller than those of the neutral PVA microgels (72.0 ± 58.3 μm). The electrostatic repulsion of ion-modified PVA also enhanced both the uniform distribution and smooth sliding of adjacent particles.

Different from support baths composed of particles, continuous-phase support baths are composed of polymer solutions with shear-thinning and self-healing properties. The common continuous phase support baths utilized are cellulose-based materials^[38,57,58]^, hyaluronic acid (HA)^[59,60]^, Pluronic F-127^[61,62]^, and Xanthan gum (XG).^[63,64]^ Additionally, silicone-based support baths have been explored for microfluidics applications.^[65,66]^ The continuous phase support baths along with their respective bioinks utilized for embedded bioprinting are listed in Table 2. The concentration or viscosity of the continuous phase support baths is a critical factor affecting the shape fidelity. For example, Shin et al.^[38]^ reported that the concentration and optimal carboxylic acid content of a cellulose nanofiber (CNF)-based support bath affect the surface roughness of bioprinted structures. As the concentration of CNF increased, the distance between cellulose molecules reduced, and the entanglement between fibers increased, resulting in higher viscosity and yield-stress, and therefore, finer bioprinted fibers. In addition, the smoothness of the printed fiber surface could also be improved with the assistance of carboxymethylation.^[38]^

Overall, either particle-based or continuous-phase support baths can be utilized for embedded bioprinting. However, a suitable support bath material needs to be selected based on the application and desired properties according to its interaction with the bioinks, as discussed in the following sections.

### 2.2. Bioinks

One of the main advantages of embedded bioprinting is that it expands the selection of bioprintable materials, especially for low-viscosity bioinks, which opens a plethora of possibilities for new applications. Hydrogels have a wide range of applications in tissue engineering, where cells can be encapsulated within hydrogels and used in embedded bioprinting to create functional constructs.^[26,67]^ Several popular hydrogels are used as bioinks, such as but not limited to gelatin, alginate, chitosan, agarose, gelatin methacrylate (GelMA), hyaluronic acid, and collagen.

For embedded bioprinting, the typical assessment method for the rheological properties of a bioink is similar to those for the conventional EBB,^[28]^ which are also the same as the support bath. For extruded bioinks, shear-thinning behavior is one of the most important rheological properties that govern the change in viscosity with applied shear stress.^[28]^ The bioink viscosity decreases at a high shear rate as shear stress induces the unraveling and stretching of chemical chains in the microstructure.^[68–70]^ During extrusion, the viscosity of a bioink influences the shear stresses applied to the encapsulated cells. It has been reported that the shear stress was at least two orders of magnitude lower for the low-viscosity bioinks (i.e., 5% w/w dextran) as compared to high-viscosity bioinks (i.e., 25% w/w Pluronic or 5% w/w alginate).^[71]^ Thus, low-viscosity bioinks could be bioprinted at a high shear rate without adversely impacting cell survival.^[68,71]^

The development of material systems in embedded bioprinting requires studying the interactions and coordination of bioinks with the support bath to ensure the structural integrity, stability, fidelity, and resolution of bioprinted constructs. The yield-stress and storage modulus of the bioink are expected to be an order of magnitude greater than those for the support bath to achieve favorable results.^[16,27,72]^ Otherwise, breakage of bioprinted fibers might occur. In contrast, the extruded fibers would be dragged along with the nozzle if they were too large compared to the support bath.^[30,72,73]^ Jin et al.^[27]^ reported that the printability of fiber was influenced by the properties of both the alginategelatin bioink and nano clay-based support bath. As the nanoclay concentration increases, low-concentration (0.5% w/v) alginategelatin fibers may be compressed due to the high storage modulus of the support bath.

In addition, the thixotropy of the bioink is also required to allow for its rapid recovery after being bioprinted into the support bath and to maintain the morphology of bioprinted fibers in the support bath until crosslinked. For temperature-sensitive bioinks, the phase change is regulated with temperature. In this case, temperature and pressure must be monitored and coordinated during bioprinting to ensure cell viability and shape fidelity.^[53]^ For instance, GelMA has a very low viscosity at 37 °C, which is cell-friendly; however, increasing its viscosity also escalates shear stresses on encapsulated cells, thus impairing cell viability.^[74]^ Therefore, embedded bioprinting, which offers bioprinting of low-viscosity bioinks, has the advantage of maintaining high cell viability.

The material system in embedded printing should be carefully selected to ensure the proper compatibility between the bioink and support bath. The ideal way to check their compatibility is to compare their rheological properties with the help of physics-based models, which are discussed in the next section.

### 2.3. Physics-Based Modeling of Rheological Behavior

The rheological properties of support baths and bioinks are often used as indicators for bioink printability and shape fidelity.^[75]^ To explore the complex rheological behavior of non-Newtonian fluids, several physics-based models are commonly applied to establish the relationship between shear stress and shear strain, such as Herschel-Bulkley (H-B), Bingham, Casson, and Carreau. Among them, the H-B model is widely used to analyze the rheological behavior of support baths because of its reasonable prediction and fitting ability.^[76]^ The H-B model equation is expressed as $\sigma =\sigma_{y}+k{\dot{\gamma }}^{n}$, where *σ*_*y*_ is the yield-stress, *k* is the flow consistency index, $\dot{\gamma }$ is the shear rate, and *n* is the flow behavior index. *k* and *n* are constant for a specific material. The yield-stress parameter captures the properties of a material at a point, where it starts to soften or yield under the applied stress. When *σ* < *σ*_*y*_, the fluid behaves as a solid, while it is a liquid otherwise. Further, if *n* < 1, the fluid experiences a decrease in viscosity with shear rate (shear-thinning), and if *n* > 1, the fluid is shear-thickening, and the viscosity increases with an increase in shear rate. Li et al.^[32]^ fitted the measured rheological data with the H-B model to quantify the yield-stress of support baths with variational compositions of PluronicF-127 and hydrophobically-modified hydroxypropylmethyl cellulose (H-HPMC). Their support bath material showed shear-thinning behavior and the yield-stress was adjustable from 1.12 to 116.44 Pa.

Bingham model follows the equation $\sigma =\sigma_{0}+k\dot{\gamma }$, and is obtained by evolving the H-B model with *n* = 1, where *k* is a constant plastic viscosity. Although the Bingham fluid model is concise, it might lead to poor accuracy because of its linear characteristics, when the yield-stress is excessive.^[29]^ Besides, the power-law model ($\tau =k{\dot{\gamma }}^{n}$) is also used to fit the measured shear stress-shear rate curve, where *k* is the power-law consistency factor and *n* is the power-law flow behavior index. Bing et al.^[77]^ used this model to analyze the rheological behavior and printability of sacrificial inks in terms of the model parameters *k* and *n*. However, it is important to note that the power-law model can only be applied to a shear rate range of 10–10^4^ s^−1^, assuming that the fluid is stable and follows a linear regime at moderate shear rates and does not account for wall sliding within the needle.^[75]^

Although the three-parameter H-B model effectively describes the yield-stress, it fails to consider the zero shear rate. In contrast, the four-parameter Carreau model estimates zero and infinite shear rate viscosity plateaus and is considered a promising model for characterizing the rheology of bioinks and suspensions.^[78]^ The Carreau model is expressed as *η*_∞_ + (*η*_∞_ + *η*_0_) × (1 + (*k* × *γ*)^2^)^(*n* − 1)/2^, where *η*_0_ describes the viscosity of the formulation at low or zero shear rate, *η*_∞_ describes the viscosity of the formulation at a high or infinite shear rate, constant *k* is equal to the reverse critical shear rate, and *n* is the fluid flow index that describes the shear shrinkage property of the formulation.^[79]^ The rheological parameters of several popular support bath materials (e.g., agarose and Carbopol) and bioinks (GelMA) have been matched with the Carreau model to effectively predict the deviation of bioprinted fibers from the intended design, quantify the bioprinting resolution, and improve the bioprinting outcomes by adjusting parameters, such as printing speed and material formulation.^[79]^

### 2.4. The Influence of Other Characteristics of Material Systems

Although bioinks and support baths are usually not mutually soluble, it is important to pay attention to their interactions. A proper pairing between the support bath and bioink reduces bioprinting defects (e.g., shape distortion and sharpening), enabling the biofabrication of high-quality structures.

#### 2.4.1. Interfacial Instability

**Figure 2.**
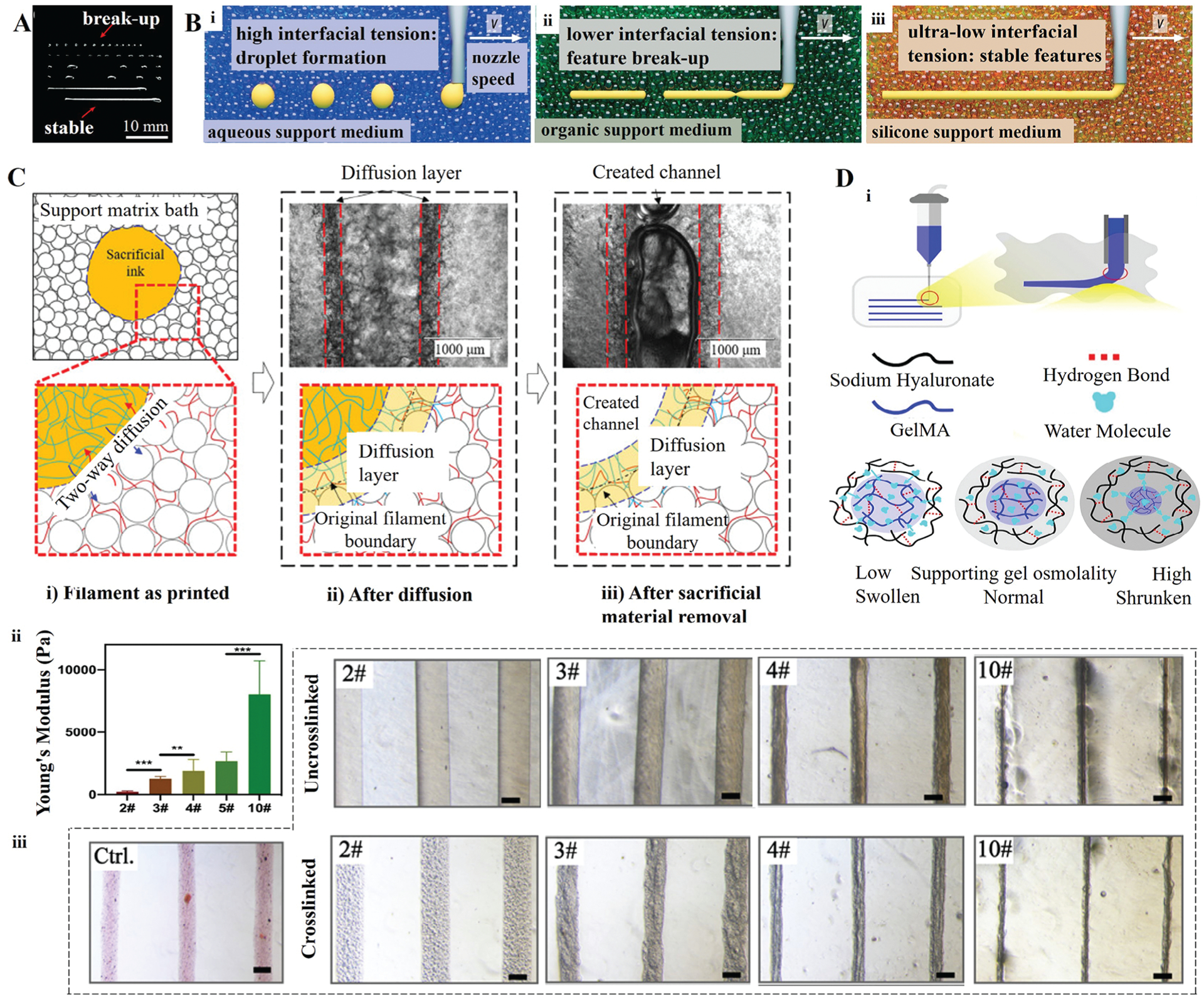
(A) Fibers with radii smaller than the critical radius broke up into smaller droplets; Adapated with permission.^[39]^ Copyright 2021, Royal Society of Chemistry. (B) Interfacial tension drives feature breakup in embedded bioprinting: (i) High interfacial tension drove them to break into spherical droplets. (ii) Intermediate interfacial tension provided some stability but limited the minimum stable feature size. (iii) Ultralow interfacial tension eliminated interfacial instabilities, removing the limits on the minimum stable feature size; Adapated with permission.^[81]^ Copyright 2023, American Association for the Advancement of Science. (C) A schematic and images showing the diffusion and channel formation that caused a fiber/channel diameter variation; Adapated with permission.^[77]^ Copyright 2022, AIP Publishing LLC. (D) IISBP (i) the schematic representation of the technique showing the fiber embedded in the HA support bath during bioprinting; (ii) Young’s modulus of the scaffolds formed by the GAH-ink in different HA support bath. (iii) Optical photographs of the fibers formed by the GAH-ink printed in different HA support baths; Adapated with permission.^[40]^ Copyright 2022, American Chemical Society.

Since a bioink is deposited inside a support bath during embedded bioprinting, interactions between the two materials are in-evitable. In particular, when the material system consists of a hydrophilic and a hydrophobic material, an interfacial tension is generated at the interface of these two materials, which negatively impacts fiber morphology, causing deformities in fibers, which may lead to disintegration of bioprinted structures.^[16,33,80]^ The Rayleigh instability interprets this phenomenon by considering the imbalance between the interfacial tension and yield-stress of the support bath, which leads to bioprinted fibers breaking into droplets. Hence, such a fiber discontinuity could be avoided by balancing the yield-stress of the support bath and bioink. O’Bryan et al.^[39]^ observed the stability of fiber bundles after bioprinting over 24 h. They discovered that fibers with a radius less than the critical value broke into smaller droplets. In contrast, fibers with a radius larger than the critical value overcame the effects of interfacial tension and maintained a stable structure (Figure 2). In addition, these measurements were repeated in support baths with a different yield-stress, and the results revealed that the minimum radius required for bioprinted fibers to remain stable was inversely proportional to the yield-stress of the support bath.^[39]^ Therefore, the stability of bioprinted fibers in the support bath is predicted from the radius (*r*) of fibers, the tension (*γ*) at the interface between the support bath and bioink, and the yield-stress (*σ*_*y*_) of the support bath, which could be formulated as *σ*_*y*_ > *γ*/*r*.^[39]^ This relationship determines the critical fiber radius (*r*_*c*_), or the smallest fiber diameter for stable bioprinting, which is *r*_*c*_ > *γ*/*σ*_*y*_. Furthermore, the introduction of new strategies reduces the impact of interface instability to obtain stable and high-quality structures. Duraivel et al.^[81]^ developed a method called additive manufacturing at ultralow interfacial tension (AMULIT), which eliminates interfacial instabilities in silicone printing (Figure 2B). The ultralow interfacial tension between the AMULIT support material and poly(dimethylsiloxane) (PDMS) inks enabled the printing of stable features with diameters as small as 8 μm. Becker et al.^[71]^ used aqueous two-phase systems (ATPS) to achieve stable printing with low-viscosity inks as the interfacial tension of ATPS (10^−4^-0.44 mN m^−1^) was drastically lower than the interfacial tension of water/air (72 mN m^−1^) or water/oil (1–40 mN m^−1^). In most biological applications, cell-laden bioinks and support baths are usually hydrophilic to establish an environment conducive to cell survival, in which case the effect of interface tension on print fidelity can be ignored.^[27]^

#### 2.4.2. Gravitational Instability

Although the support bath provides in situ self-support for the bioink, it is vital to balance the density between the bioink and support bath to maintain the stability of bioprinted structures. In this regard, Luo et al.^[82]^ reported that structures bioprinted with a material system of dextran-polyacrylamide/poly(ethylene oxide) DEX-PAAm/PEO-2m (Mw = 2000000) remained stable for a pro-longed time than those bioprinted with a DEX-PAAm/PEO-8k (Mw = 8000) system. The PEO-8k support bath had a low zero-shear viscosity (0.25 Pa·s), and the density difference between this support material and the bioink was 50 kg·m^−3^, where bioprinted structures sank quickly. In contrast, PEO-2m showed a greater zero-shear viscosity (38 Pa·s) and a smaller density difference (10 kg·m^−3^), where bioprinted structures remained stable in the vertical direction and retained their shape for up to several days without cross–linking of bulk liquid phases. Becker et al.^[71]^ further explored the effects of ink/slurry density mismatch on the sedimentation speed of polyethylene glycol (PEG) droplets. The results revealed that during bioprinting in a dextran-based bath with varying concentrations, sedimentation speed was positively correlated with density mismatch and inversely correlated with the bath viscosity. The stability condition can be expressed by the equation Δ*ρVg* < *σ*_*y*_*A*_*h*_, where Δ*ρ* is the density difference between the support material and bioink, *V* is the volume of the bioink, *g* is the acceleration of gravity, *σ*_*y*_ is the yield-stress of the support material, and *A*_*h*_ is the surface area of the bioink. Therefore, matching the density of the bioink and support material reduces the instability resulting from the buoyancy force.^[29,33,80]^

#### 2.4.3. Osmotic Pressure Difference

Osmotic pressure differences can drive solvent exchange between a bioink and support bath from the location with a lower concentration to that with a higher concentration.^[40,83,84]^ In this situation, the osmotic pressure is the driving force behind the bioink diffusion, which causes a change in the diameter of bioprinted fibers.^[40]^ In particular, diffusion limits the resolution when a bioink with extremely low viscosity is bioprinted. It has been reported that the diffusion time of a low-viscosity dye fiber from the fiber side to the agarose support bath side was over two hours.^[47]^ The results revealed that the lower the viscosity the ink possessed, the more pronounced the diffusion was, and smaller fiber gaps were observed with time, which led to inferior resolution and fidelity.^[36]^ Ren et al.^[77]^ discovered bi-directional diffusion between a hydrophilic sacrificial ink and a gelatin support bath, as shown in Figure 2C. After bioprinting, the deposited sacrificial ink diffused into the support bath, while the gelatin precursor of the support bath diffused into the sacrificial ink. As a result, the mechanical strength and integrity of fibers diminished due to diffusion. Further, depending on the diffusion degree, if the resultant fibers retain enough mechanical strength to survive during the removal process, it gives the final channel diameter lesser than the design diameter. Therefore, choosing a material system with a small diffusion coefficient and a method, where printed fibers can be crosslinked quickly minimizes the impact on print resolution and fidelity.^[77]^ The increase in bioprinted fiber’ diameter caused by ink diffusion can be predicted by Fick’s second law.^[16]^ In addition, controlling the regulation of the rheological characteristics of the support bath alleviates the diffusion issue. To prevent osmotic exchange between the bioink and support bath, Rahimnejad et al.^[36]^ added 1% sodium chloride to the Pluronic support bath, effectively providing an osmotic pressure molar concentration to the support bath comparable to that of the bioink.

Although diffusion of materials usually affects bioprinted structures, osmotic pressure can also be utilized to modulate the properties of bioprinted fibers, including fiber size, pore structure, mechanical strength, and water absorption.^[40]^ Wang et al.^[40]^ reported an infiltration-induced suspension bioprinting (IISBP) technique based on a HA support bath (**2**D). For a non-ideal dilute solution, the theoretical osmotic pressure can be described by the Virial equation, $\frac{\Pi }{\rho }=RT\left(\frac{1}{M}+A_{2}\rho +A_{3}\rho^{2}+\cdots \right)$, where Π is the osmotic pressure, *ρ* is the mass concentration of the solute, *M* is the molar mass of the solute, *R* is the molar gas constant, *T* is the Kelvin temperature, and *A* is the virial coefficient. After bioprinting, the osmotic pressure difference between the bioink and support bath caused fibers to absorb or exude the solvent, resulting in their expansion or shrinkage (Figure 2D). Overall, as the concentration of the HA support bath increased, the diameters of bioprinted fibers decreased significantly. Also, the results exhibited that the IISBP technique was able to control the resolution of bioprinted fibers in the range of 37.5–169.2% (compared to the control group).

## 3. Printing Parameters

In addition to material systems, the print fidelity and resolution are equally affected by the printing parameters, which can be divided into two categories, namely process parameters (e.g., nozzle size, printing speed, and flow rate) and design parameters (e.g., tool path planning and inter-fiber spacing).

### 3.1. Process Parameters

#### 3.1.1. Nozzle Size

**Figure 3.**
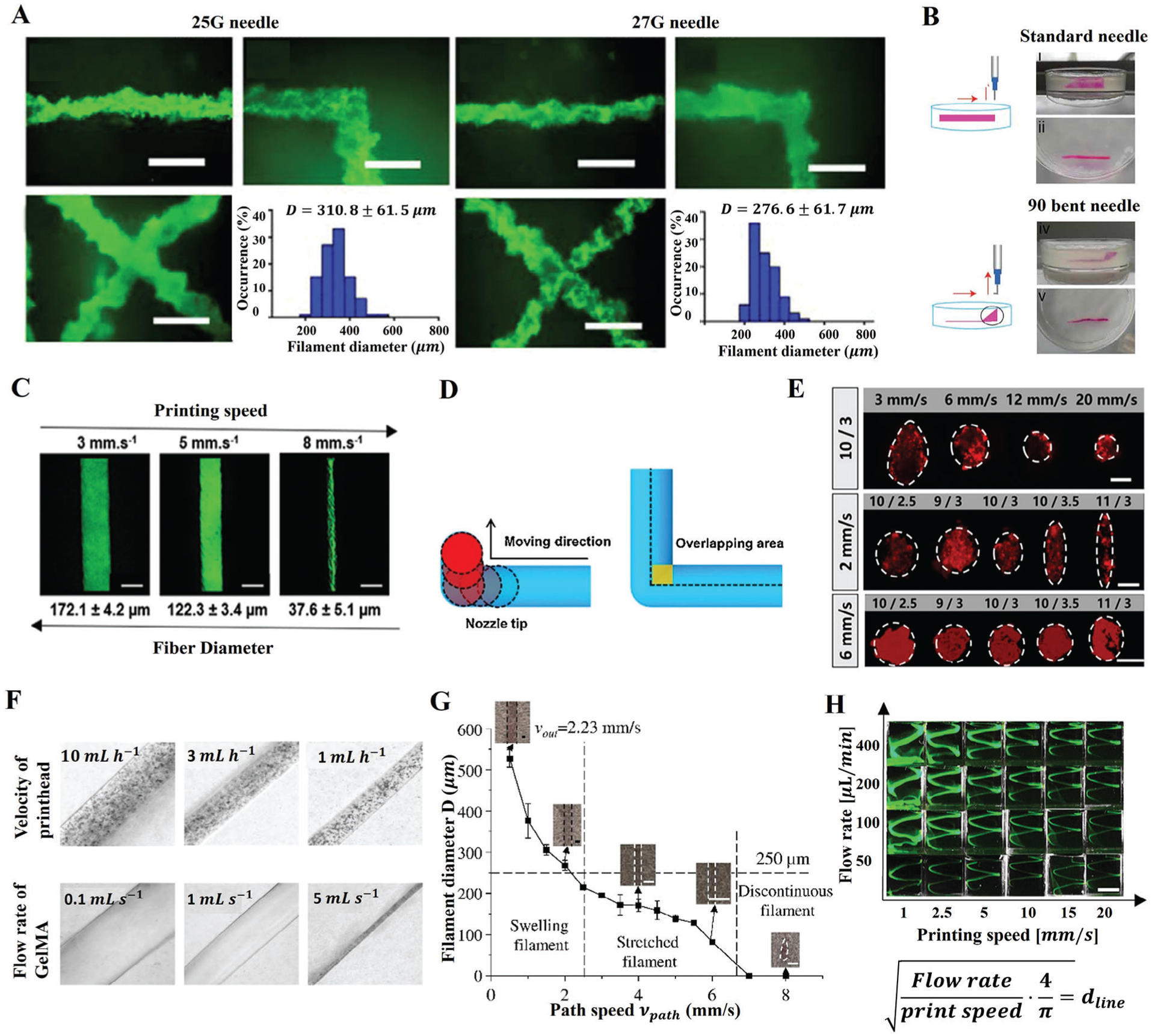
A) LIVE/DEAD staining and diameter distribution of hMSC-laden fibers with various configurations, which were bioprinted into a support bath of alginate microgels using 25 and 27 G nozzles (scale bar = 600 mm); Adapated with permission.^[54]^ Copyright 2019, Royal Society of Chemistry. B) Bioprinting Newtonian fluids in a Xanthan gum bath using a straight and 90° bent nozzle; Adapated with permission.^[63]^ Copyright 2022, IOP Publishing. C) Resolution of fluorescein-isothio-cyanate (FITC)-labeled gelatin bioink bioprinted at a constant pressure and varied printing speed (scale bar = 150 μm); Adapated with permission.^[95]^ Copyright 2021, Wiley-VCH. D) Formation of an overlapping area during bioprinting a fiber with a right angle; Adapated with permission.^[38]^ Copyright 2021, Elsevier. E) Cross-sections of fibers that were bioprinted at various printing speeds (scale bar = 200 μm); Adapated with permission.^[32]^ Copyright 2022, American Chemical Society. F) Microscopic images of GelMA fibers bioprinted with different flow rates and printing speed; Adapated with permission.^[96]^ Copyright 2023, National Academy of Sciences. G) Effects of *V*_path_ and *V*_out_ on fiber formation; Adapated with permission.^[86]^ Copyright 2017, Elsevier. H) False-colored spiral prints were obtained with varying printing speeds and flow rates; Adapated with permission.^[71]^ Copyright 2023, Wiley-VCH.

In embedded bioprinting, nozzle diameter is one of the major parameters that determine the resolution of bioprinted features. The use of a nozzle with a large diameter increases the layer height and fiber width due to the increased volumetric flow rate of extruded bioink, subsequently decreasing the resolution.^[18,19,31,63,85–87]^ Jeon et al.^[54]^ bioprinted human mesenchymal stem cell (hMSC) aggregates into a support bath of alginate microgel using a 27G nozzle, where a smaller fiber diameter was obtained using a 25G nozzle. Further, the thinner nozzle demonstrated lesser shape distortion of fibers when bioprinting multiple or intersecting lines since a smaller volume of gel inside the bath was disturbed during nozzle movement (Figure 3A).^[54]^ A similar investigation was conducted by Jessica et al.,^[47]^ in which an agarose support bath was utilized, and it was reported that the fiber size and distortion, the area of disturbance, and the size of cracks increased with a greater nozzle size. In addition, the nozzle shape also affects the process stability. Trikalitis et al.^[63]^ used a standard 21G straight nozzle and a 90° curved nozzle to bioprint cell suspensions in a Xanthan gum support bath. The use of a curved nozzle prevented the flow disturbance of the bioink around the nozzle, as shown in Figure 3B. Furthermore, during extrusion, shear stress is generated inside the nozzle, which can impair cell viability, and the shear stress that is exerted on cells varies depending on the shape and size of the nozzle. Studies have shown that conical and straight nozzles also affect cell viability. Straight nozzles apply lower stresses at the exit, and conical nozzles, on the contrary, show higher stresses at the exit, producing a greater area of perturbation.^[53,88–92]^ Further, a straight nozzle is more suitable for embedded bioprinting than a conical nozzle because the latter displaces larger volumes of gel during its movement owing to its conical design. Besides that, the shear stress exerted on cells increases with decreasing nozzle size during extrusion.^[88,93,94]^ A nozzle with a smaller diameter requires higher pressure than a larger diameter nozzle to maintain the same flow rate leading to increasing shear and extensional stresses borne by cells. Thus, it is essential to consider the shape and size of the nozzle and the bioink flow rate to achieve high printing resolution and cellular viability simultaneously.

#### 3.1.2. Printing Speed

The speed of the nozzle movement has also an important role in the print resolution and fidelity. Keeping the other parameters constant, Bakht et al.^[95]^ investigated the effect of printing speed on fiber size. The gelatin fiber size could be easily tuned by varying the printing speed ranging from 172 μm at 3 mm.s^−1^ down to 37 μm at 8 mm.s^−1^ at a constant pressure of 5 kPa with a 30G nozzle (Figure 3C). An exceptionally wide range of fiber diameters could be printed using a single nozzle diameter by simply varying the printing speed.^[71,95]^ In addition to improving resolution, adjusting printing speed improves structural fidelity, such as printing accurate angle and diameter uniformity throughout the fiber length. There were overlapping areas at the intersection of horizontal and vertical fiber when a structure with right angles was bioprinted (Figure 3D). In such a process, bioprinted features may be deformed due to the increased volume of extruded bioink at the angle. Shin et al.^[38]^ controlled the printing speed to maintain the volume of extruded bioink to reduce the difference between the printed and designed angles. Though increasing the printing speed was favorable to forming well-defined fibers, a further increase in the printing speed would result in discontinuities in printed fibers. A higher printing speed might enhance the drag force exerted on the deposited bioink, causing the printed fibers to break. Further, a higher printing speed also causes a higher level of shear stress on the surrounding bath that liquefies the material nearby, which further makes the fiber move and break into segments.^[32,38]^ During high-speed bioprinting, bubbles may be introduced into the support bath along the printing path, which affects the bioprinting process and constrains the printing speed.^[96]^

Since the nozzle moves through the support bath, a well-defined fiber morphology could be obtained by balancing printing speed and rheological properties. A comprehensive list of parameters (i.e., printing speed, nozzle size, etc.) utilized in embedded bioprinting is tabulated in Table 3. By analyzing the continuity and crosssection of bioprinted fibers, Li et al.^[32]^ explored the coupling effect of the printing speed and yield-stress of the support bath. An increase in printing speed from 3 to 20 mm ^−1^s altered the cross–section from elliptical to circular and resulted in decreased fiber diameter, while an increase in viscosity made the cross–section more elliptic (Figure 3E). Increasing the printing speed and the yield-stress of the support bath favored the formation of fibers with widths equal to or less than the nozzle size to improve printing resolution. Meanwhile, improving the yield-stress could prevent the liquefaction of the bath and strengthen the friction force to ensure printing continuity.^[32]^ While increasing the printing speed, the competition between inertial and viscous forces causes Reynold’s instability, which might induce an air gap in the wake of the printing nozzle.^[97]^ To avoid the instability, it can be tuned using Reynold’s number, which is defined as *Re* = *ρυd*∕*η*, where *ρ* is the density of the bioink, *η* is the viscosity of the bioink, *d* is the diameter of the printing nozzle, and *υ* is the printing speed. The study results exhibited that wake recirculation began to occur at *Re* = 10–15. For higher printing speeds, Reynold’s instability can be avoided by adjusting the nozzle diameter and ink viscosity.^[33,80]^

#### 3.1.3. Flow Rate

The flow rate of the bioink can also affect the resolution of printed fibers. By approximating the cross–section as a circle, the diameter of bioprinted fibers can be predicted by the law of conservation of mass^[39]^: $r=\sqrt{4Q/\pi v}$, where *Q* is the extrusion flow rate, *υ* is the printing speed, and *r* is the radius of the printed fiber. The line width (2*r*) is proportional to the flow rate and inversely related to the printing speed (*υ*). For example, diameters of GelMA fibers (from 10 to 700 μm) could be readily controlled by changing the flow rate of GelMA and the printing speed (Figure 3F).^[96]^ Jin et al.^[86]^ explored the competition between printing speed and flow rate (*V*_*path*_/*V*_*out*_) to prevent irregular morphology and un-controllable diameter of fibers. When *V*_*path*_ is equal to *V*_*out*_, the volume of extruded bioink forms a fiber with a diameter equal to the nozzle diameter. If *V*_*path*_ < *V*_*out*_, excessive bioink deposition leads to the thicker fibers. If *V*_*path*_ > *V*_*out*_, a stretched fiber is extruded with a diameter smaller than the diameter of the nozzle. When *V*_*path*_ increases further, discontinuties can be observed (Figure 3G). Based on the flow rate and printing speed, the diameter of bioprinted fibers could be calculated. Spiral patterns of different thicknesses were bioprinted by simply varying the *V*_*out*_ and *V*_*path*_ (Figure 3H).^[71]^

The flow rate can be modulated by controlling the extrusion pressure. For different types of bioinks, the increase in the extrusion pressure results in different changes in fiber diameters. Ren et al.^[77]^ described the variation of fiber diameter with respect to the change of applied pressure. For instance, fiber diameter increased > 2.5 times for Pluronic when the air pressure rose from 60 to 85 kPa. However, there was only a slight increase in the fiber diameter under the same circumstances for PVA. When the nozzle diameter and printing speed are maintained constant, the fiber diameter can be predicted by the pressure drop using the equation of *d*_*f*_ ~ (Δ*p*)^1∕*n*^, where *n* is the flow behavior index.^[77]^ Additionally, the relationship of *n* with the feature diameter sensitivity parameter (*K*) can be approximated by the equation of *K* = 14.4 − 14.0 × *n*. Thus, the shear-thinning bioinks (*n* < *1*) are more sensitive to pressure.^[98]^

### 3.2. Design Parameters

#### 3.2.1. Path Planning

**Figure 4.**
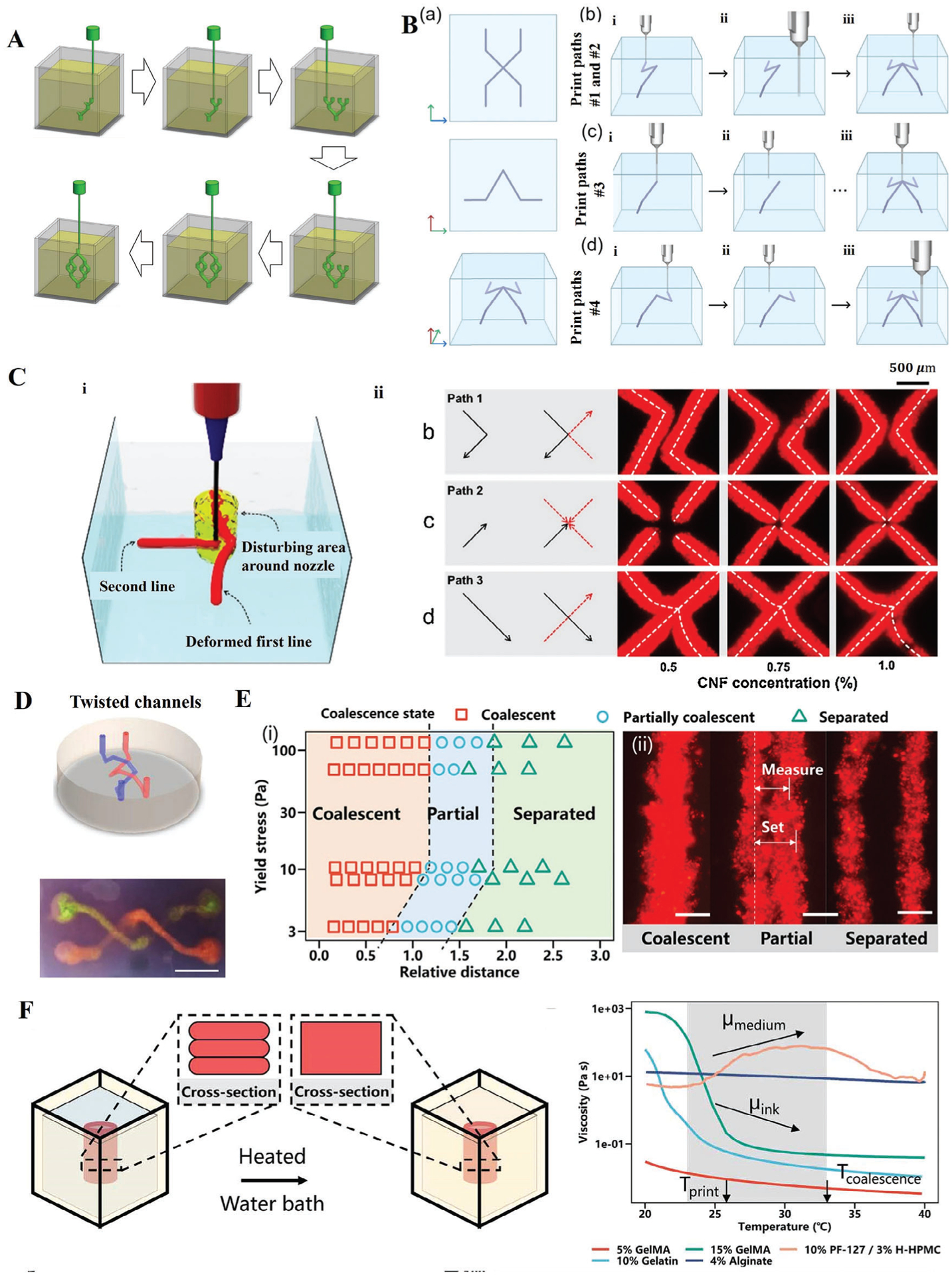
A) A “localized layer-by-layer” bioprinting approach to fabricate cell-laden microvascular network; Adapated with permission.^[99]^ Copyright 2017, American Chemical Society. B) Effects of the composition of the support bath and print path on fidelity; Adapated with permission.^[73]^ Copyright 2018, American Chemical Society. C) (i) A schematic illustration showing feature deformation due to the disturbance caused by the movement of the printing nozzle. (ii) Three paths for printing the “X” pattern to evaluate the effect of path plans and bioink concentrations on the print fidelity; Adapated with permission.^[38]^ Copyright 2021, Elsevier. D) Biorinted twisted channels with a z-offset at the path crossing in a gelatin-gellan support bath (scale bars = 5 mm) ; Adapated with permission.^[51]^ Copyright 2020, American Chemical Society. E) (i) Phase diagram about the coalescence state between adjacent fibers based on the yield-stress and relative distance. (ii) Representative coalescence states between adjacent fibers (scale bars = 200 μm); Adapated with permission.^[32]^ Copyright 2022, American Chemical Society. F) A schematic illustration of the EAW method and the thermoresponsiveness of the support bath and bioink; Adapated with permission.^[37]^ Copyright 2022, IOP Publishing.

Embedded bioprinting has expanded its ability to imitate the complex geometry of native tissue. Unlike the conventional strategy of designing paths layer-by-layer, embedded printing has the advantage of omnidirectional printing, which allows for free movement of the nozzle in any direction in the support bath and rapid printing of complex path networks. Thus embedded bioprinting supports path planning with more degrees of freedom, thereby offering a more efficient travel path with better print quality. For example, Jin et al.^[99]^ provided a ″localized layer-by-layer″ bioprinting procedure to generate a cell-laden microvascular network (Figure 4A). This method improved the bioprinting efficiency of large spanning structures by minimizing unnecessary discontinuities in bioink deposition. Moreover, Grosskopf et al.^[73]^ investigated how the path plan influences pattern fidelity. As shown in Figure 4B, different paths can lead to deviations in the position of fibers. Independent of the composition of the support bath, optimization of the path plan facilitates the generation of patterns that are most similar to the design. In addition, to investigate the effect of various printing paths on the bioprinted structure, Shin et al.^[38]^ presented several path plans to print the “X” pattern using the same printing parameters (Figure 4C). Corners and junctions tend to be distorted when printed at higher speeds or when the path plan is such that the nozzle crosses an existing fiber. This is because the nozzle movement induces shear stress to the gel which is higher than its yield-stress, causing the previously deposited ink to split and deform.^[51]^ Compaan et al.^[51]^ solved this problem by offsetting path crossings slightly in the z direction and extending the bioprinted fiber for 0.5 mm beyond the intersection point, which is an effective approach to create a twisted channel with excellent connectivity (Figure 4D). By advancing path planning for complex structures with hundreds of fibers, Weeks et al.^[100]^ developed a graph theory-based algorithm that automated bioprinting of periodic and stochastic architected lattices. This algorithm took advantage of embedded bioprinting to directly deposit a fiber in 3D instead of slicing it into numerous layers.

#### 3.2.2. Inter-Fiber Spacing

In order to print a fiber next to an existing fiber to fabricate a structure with thicker walls, it is necessary to ensure the coalescence between adjacent fibers. It is essential to acquire a strong bonding among the neighboring fibers to guarantee the fidelity and stability of final structures. Three fiber aggregation types exist, namely coalescent, partially coalescent, and separated (Figure 4E). Large gaps between neighboring fibers cause bioprinted fibers to detach from one another, impairing the structural integrity. Conversely, coalescence happens when the space between adjacent fibers is smaller than the fiber diameter. The nozzle that moves quickly reduces the local pressure, which causes pressure disturbance and encourages the aggregation of adjacent fibers, following Bernoulli’s principle.^[32]^ It has also been proven that a reasonable inter-fiber distance and a support bath with relatively high yield-stress are beneficial for the coalescence of adjacent fibers, ensuring the stability and fidelity of bioprinted structures.^[32]^ Moreover, to enhance the bonding of adjacent layers after extrusion, Li et al.^[37]^ introduced a versatile method, called enhanced adhesion writing (EAW), and the results showed promising printability, shape fidelity, and mechanical strength. EAW utilized the thermoresponsive rheological properties of the support bath and bioink between 22 °C and 33 °C (Figure 4F). As the temperature rises, the viscosity of temperature-sensitive bioinks declines while the viscosity and yield-stress of the support bath increase, facilitating the bonding of neighboring layers, keeping the extruded bioinks in position, and preventing their diffusion.

## 4. Embedded Bioprinting of 3D Complex Structures

### 4.1. Cross–linking Mechanisms

After the bioink extrusion, cross–linking is necessary to maintain the bioprinted structures in the target position. Cross–linking refers to the process of connecting functional groups or reactive sites within molecules or polymers together under chemical or physical conditions, forming a 3D network through chemical bonds, physical forces, or ion exchange. Cross–linking can make molecules or polymers more stable and robust in morphology, increasing their mechanical strength and durability. In embedded bioprinting, methods with the involvement of ultraviolet (UV) light, ions, or enzymes are commonly used to induce or promote cross–linking reactions in bioinks, thereby forming tissue constructs with stable structures after bioprinting.^[101]^

Photocrosslinking is a flexible cross–linking mechanism and the popular photocrosslinkable hydrogels include GelMA, methacrylated hyaluronic acid (HAMA), norbornene-modified hyaluronic acid (NorHA), poly(ethylene glycol) diacrylate (PEDGA) and silk fibroin (SF), which can be crosslinked with the assistance of photoinitiators.^[102–106]^ It is well known that the intensity and exposure duration of UV light play crucial roles in the stiffness of crosslinked polymers. Stronger hydrogel structures are produced by higher UV intensities and longer exposure time, which, however, hinders biological functions such as cell spreading and migration due to the densely packed molecular network. In addition, although UV light can easily penetrate through the support bath to cure the bioink, the luminous intensity gets attenuated in deep regions of bioprinted constructs post-bioprinting, resulting in a reduction in stiffness. Ning et al.^[26]^ described the impact of UV light passing through a carborundum-based support bath and discovered that the stiffness of GelMA, which crosslinked in the support bath, was lower than that crosslinked in the air. Furthermore, the diameter of bioprinted fibers may change as a result of photocrosslinking. Wang et al.^[40]^ covalently crosslinked GAH ink (GelMA-Acrylamide-HA), which led to a decrease in fiber diameter and an improvement in printing resolution, and at the same time, bioprinted fibers exhibited a rough appearance because of drying during cross–linking.

Ionic cross–linking occurs through the formation of electrostatic bonds between ions and polymer chains with opposite charges, which retain bioprinted structures upon cross–linking.^[107]^ Alginate is a well-known example of ionic cross–linking when exposed to divalent cations, such as calcium ions. Adult human heart models of alginate have been generated by applying the FRESH method, which was ionically crosslinked with a calcium chloride solution. Dimensions of bioprinted structures were calculated and found comparable to the original design.^[19]^ Furthermore, the addition of calcium ions hardens the hydrogel particles, leading to an increase in yield-stress of the particle-based support bath.^[51]^

Enzymatic cross–linking can also enhance the intermolecular connections at an optimal enzyme activity.^[107]^ Melo et al.^[108,109]^ used thrombin as a catalyst to trigger the covalent connection of fibrinogen to form stable fibrin hydrogel, creating a soft environment suitable for chondrocyte survival. Furthermore, gelatin can also be chemically crosslinked using enzyme agents, such as transglutaminases (TGs), which covalently connect gelatin molecules to form networks. For example, Ashley et al.^[51]^ converted the gelatin support bath into crosslinked hydrogel with the help of TGs after bioprinting, and the sacrificial ink was directly removed to form channels in bulk hydrogel. However, it should be noted that for cell-laden constructs, the culture medium may deactivate TG. A comprehensive list of reported articles with different cross–linking mechanisms is tabulated in Table 4.

In addition, unlike the above-mentioned cross–linking mechanism that needs external reagents, the Schiff-base cross–linking can be applied for interactions between free amino and aldehyde groups in the extruded bioink and support bath. For example, Heo et al.^[110]^ bioprinted carbohydrazide-modified gelatin (Gel-CDH) into a support bath consisting of gelatin microparticles suspended in an oxidized alginate (OAlg) solution. Schiff base reaction facilitated the formation of gelatin-based fibers via the imine bond between aldehyde groups of OAlg and amino groups of Gel-CDH. For maintaining the position and integrity of extruded fibers, the cross–linking time and the degree of cross–linking could be modified by changing the concentration of OAlg and Gel-CDH.

### 4.2. Customization of Embedded Bioprinting For Tissue Reconstructions

Through the investigation of material systems and printing parameters during embedded bioprinting, successful outcomes with bioprinting of tissues and organs have been obtained as well. Influences of critical factors on the resultant constructs are discussed in this section, by taking some applications as examples, such as vascular channels, heart, cartilage, etc.

#### 4.2.1. Vascular Channels

The generation of vascular networks is one of the most prominent applications of embedded bioprinting. Blood vessels play a vital role in human tissues and organs, delivering oxygen and nutrients to cells.^[31,111]^ However, there exist several challenges in developing vascularized tissues via bioprinting including maintaining long-term retention and integrity of vasculature during/post bioprinting and *in-situ* endothelialization of bioprinted constructs. Embedded bioprinting provides a new strategy for vascularization in tissue engineering,^[31]^ enabling bioprinting of perfusable channels, tubular structures, and vascular networks using sacrificial inks.^[53,112]^

**Figure 5.**
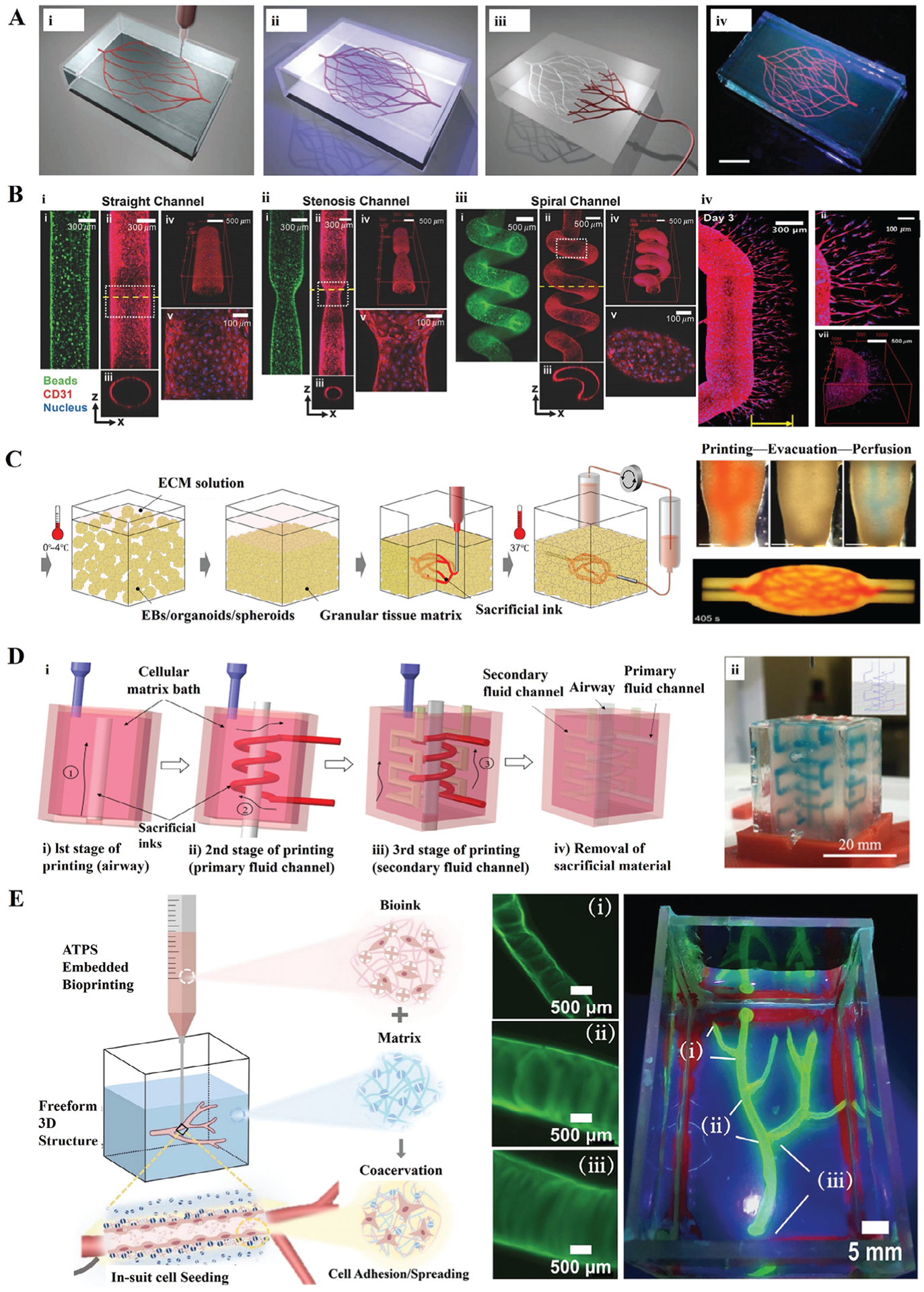
A) Schematics of embedded bioprinting of 3D microvascular networks: (i) Embedded printing of sacrificial ink allows the formation of branched networks, (ii) photocrosslinking of the support bath, (iii) liquification and removal of the sacrificial ink under modest vacuum to develop microvascular channels, (iv) fluorescent images of bioprinted constructs; Adapated with permission.^[22]^ Copyright 2011, Wiley-VCH. B) Complex-shaped printed microchannels within cell-degradable hydrogels: (i-iii) endothelial cell-seeded straight, stenosis, and spiral microchannels, (iv) angiogenic sprouting occurred from the microchannel into the support bath after 3 days; Adapated with permission.^[113]^ Copyright 2018, Wiley-VCH. C) Illustration of the SWIFT process and bioprinted branched vascular channels; Adapated with permission.^[23]^ Copyright 2019, American Association for the Advancement of Science. D) Fabrication of a perfusable airway tissue model: (i) the schematics of the embedded bioprinting process, (ii) the printed channel patterns (the sacrificial ink was dyed in blue); Adapated with permission.^[77]^ Copyright 2022, AIP Publishing LLC. E) Illustration of the workflow of the aqueous two-phase systems (ATPS) and fluorescent image of a bioprinted branched vessel network; Adapated with permission.^[114]^ Copyright 2023, Wiley-VCH.

In this regard, Wu et al.^[22]^ embedded Pluronic in a support bath of photopolymerizable Pluronic-diacrylate and the 3D microvascular networks were obtained by liquifying and removing Pluronic after photocrosslinking (Figure 5A). The authors investigated the printing behavior and rheological properties of the sacrificial ink, support bath, and fluid filler over a range of compositions to determine their respective optimal formulations. Through parameter adjustment, microchannel diameters ranging from 200–600 μm were determined. In addition to creating channels with different diameters, channels with varied shapes were also manufactured. Song et al.^[113]^ used the modified hydrogels to fabricate microchannels with three different shapes (straight, stenosis, and spiral) (Figure 5B). During fabrication, the sacrificial ink was composed of adamantane-modified hyaluronic acid (Ad-HA) and *β*-cyclodextrin modified HA (CD-HA). At the same time, the support bath was a mixture of norbornene-modified Ad-HA and CD-HA. The interaction of hydrogels maintained the quality of the printed structures and limited diffusion between the ink and the support materials. Additionally, the hydrogel formulation (e.g., bioink and crosslinker concentrations) could be altered to adjust the mechanical properties of printed structures. After seeding with human umbilical vein endothelial cells (HUVECs), the inner surface of microchannels formed a confluent HUVEC layer, mimicking the endothelium of blood vessels. Moreover, when HUVECs were exposed to angiogenic factors, they could degrade the support bath materials with proteases and form sprouts into the support bath (Figure 5B).

Instead of using hydrogel as a support bath, Skylar-Scott et al.^[23]^ bioprinted a branched and hierarchical channel network in a cellular support bath composed of induced pluripotent stem cells (iPSC)-derived organ building blocks (OBBs) (Figure 5C). By adjusting the rheology of the sacrificial gelatin ink, the sacrificial ink could be embedded in the cellular support bath and removed by warming at 37 °C. The printing resolution depended on the diameters of OBBs (200 μm) and channels with different diameters (400–1000 μm) were obtained by controlling the flow rate and printing speed. The sacrificial gelatin ink could be embedded in the cellular support bath and removed by warming at 37 °C. When HUVECs were introduced, endothelialized channels could be formed inside the constructs. These perfusable tissues also promoted the circulation of oxygenated media throughout the vascular channels and enhanced cell viability.

In addition, Ren et al.^[77]^ fabricated a tissue model with multiple channels to simulate airways (Figure 5D). To ensure the printability of channels, they chose a sacrificial ink with a low flow behavior index and diffusion coefficient. Airway structures were created using Pluronic as a sacrificial ink in a gelatin-based composite support bath with 3T3 cells, followed by cross–linking the support bath through the enzymatic action of TG and removing the sacrificial ink. Besides, the continuous perfusion of channels significantly increased the viability of cells compared to the non-perfused group, highlighting the efficacy of perfusable channels for supporting scalable tissue structures. Zhang et al.^[114]^ adopted the interfacial coacervation of the aqueous two-phase system (ATPS) in embedded bioprinting, which produced endothelialized vascular networks without any help of adhesive peptides (Figure 5E). They used dextran (DEX), polylysine (PLL), and living cells as a bioink, and an aqueous mixture of oxidized bacteria celluloses (oxBC) nanofibrils and polyethylene glycol (PEG) as a support bath. Therefore, PLL/oxBC coacervated complex could form a robust tubular structure, whose thickness and porosity varied by tuning the concentrations of PLL and oxBC, respectively. As the cell-laden PLL bioink was deposited into the oxBC medium phase, the ATPS formed filamentous scaffolds for cell attachment, spreading, and growth, which showed a functional endothelial monolayer after three days of culture.

#### 4.2.2. Heart Model

**Figure 6.**
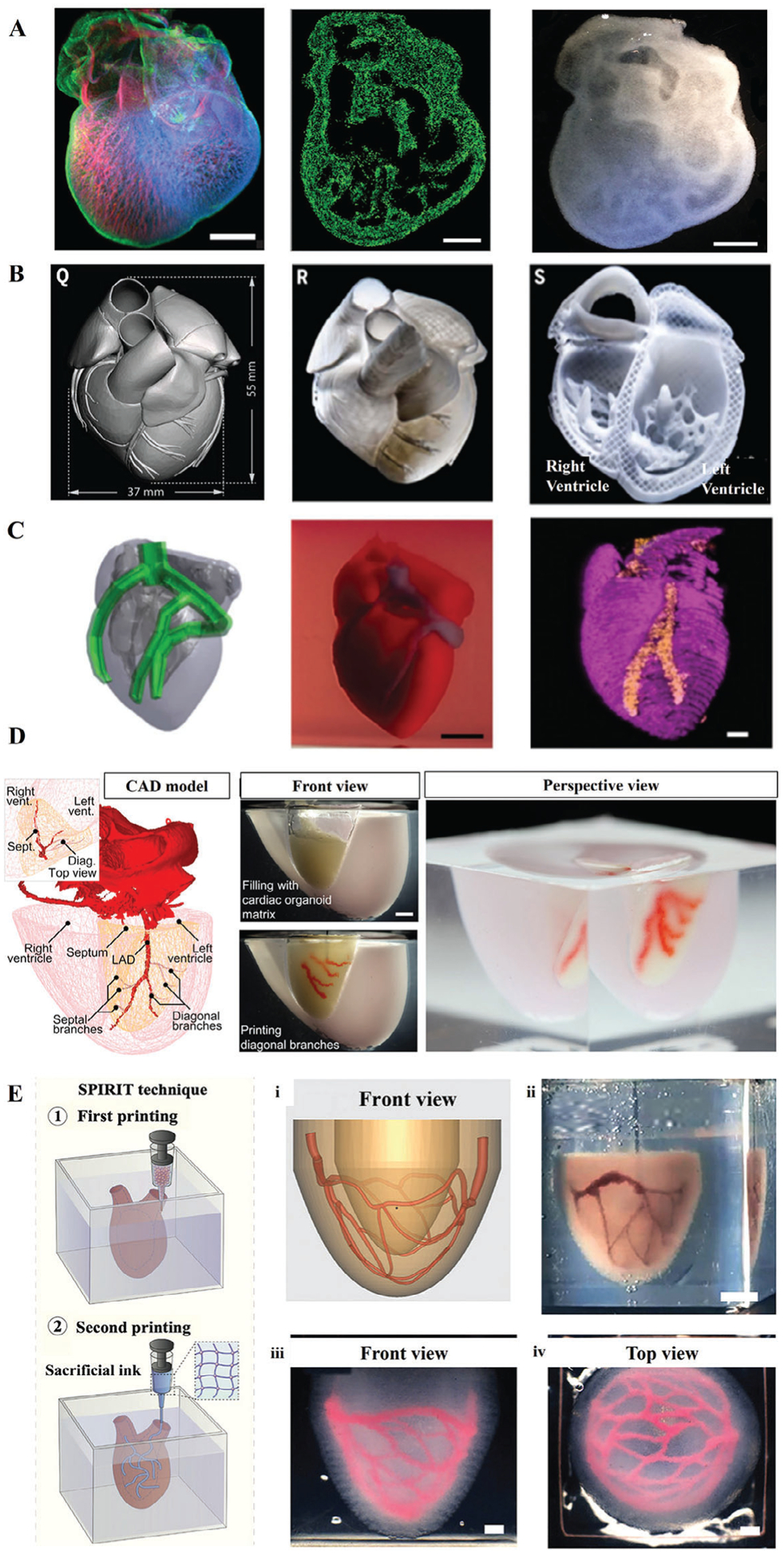
A) A 3D image of a 5-day-old embryonic chick heart (left); a cross–section image (middle) and a dark-field image (right) of the 3D printed heart; Adapated with permission.^[24]^ Copyright 2015, American Association for the Advancement of Science. B) The designed heart model (left); the side (middle) and cross–sectional view (right) of the bioprinted heart model; Adapated with permission.^[18]^ Copyright 2019, American Association for the Advancement of Science. C) Images of the magnetic resonance imaging (MRI)-derived 3D human heart (left), FRESH-printed heart (middle), and cross–sectional view of the heart (right); Adapated with permission.^[210]^ Copyright 2019, Wiley-VCH. D) A 3D model of a normal human heart and vascular branches that were bioprinted into a mode of septal-anterior wall wedge; Adapated with permission.^[23]^ Copyright 2019, American Association for the Advancement of Science. E) Perfusable ventricle constructs fabricated by SPIRIT: (i) Front view of a 3D model of the hierarchical vascular network within a ventricle construct. (ii) Extruded gelatin ink into the bioprinted ventricle. (iii–iv) The optical images showing a 3D-printed ventricle with a densely packed vascular network (indicated by red dye) at the front view (iii) and top view (iv); Adapated with permission.^[20]^ Copyright 2023, Wiley-VCH.

Over the past few years, embedded bioprinting has been shown to be a promising technique for creating organ models, particularly cardiac models. In this regard, Hinton et al.^[24]^ successfully printed a chick heart model with complex internal trabeculation using an alginate-based bioink bioprinted into a gelatin support bath (Figure 6A). In a follow-up by the same group study, Lee et al.^[18]^ optimized the support bath with microparticles and increased printing resolution to build a 3D human heart model, which included atria and ventricles, trabeculae, pulmonary artery, and aortic valve (Figure 6B). This technique extended the feasibility of bioprinting heart models from mechanically-weak materials, such as collagen. To demonstrate the capabilities of the FRESH platform in fabrication of full-size organ models, Mirdamadi et al.^[19]^ further improved the printer performance and increased the print scale in the Z axis. In this study, alginate was used to 3D bioprint a full-size model of an adult human heart that contained complex internal architecture including pulmonary and aortic valves, papillary muscle, and trabeculae carneae.

Moreover, customized bioinks and printing strategies have been developed to support the free-form printing of heart models. Noor et al.^[115]^ utilized decellularized omentum gel as a bioink and bioprinted thick, vascularized, and perfusable cardiac patches in a support bath consisting of alginate and Xanthan gum. The results demonstrated that transplanted cardiac patches contained elongated cardiomyocytes with abundant striations of sarcomeric proteins. Furthermore, as a proof of concept, a cellularized human heart with a natural structure was also bioprinted, showcasing the potential applications of this method (Figure 6C). Lee et al.^[116]^ utilized GelMA supplemented with recombinant human tropoelastin as a bioink to manufacture vascularized cardiac constructs. The extrusion pressure and printing speed were modified to achieve mechanically-robust structures. The printed structure demonstrated endothelial barrier functionality and spontaneous beating of cardiomyocytes. Upon subcutaneous implantation in rats, bioprinted constructs elicited minimal inflammatory responses and were effectively biodegradable in vivo. Skylar-Scott et al.^[23]^ used SWIFT to replicate the geometry of the arterial vascular network in the cardiac OBB matrix (Figure 6D), where the cardiac tissue matrix was monitored for its spontaneous contraction and beating. Fang et al.^[20]^ developed a process termed sequential printing in a reversible ink template (SPIRIT), where a microgel-based biphasic (MB) bioink could be used as a bioink and a support bath (Figure 6E). By optimizing the preparation process and material composition, soft structures with structural integrity were fabricated. The MB bioink loaded with neonatal rat ventricular cardiomyocytes (NRVC) was prepared to bioprint ventricular models with perfusable vascular networks, which significantly enhanced the viability of bioprinted tissues during in vitro culture.

#### 4.2.3. Other Functional Tissues

**Figure 7.**
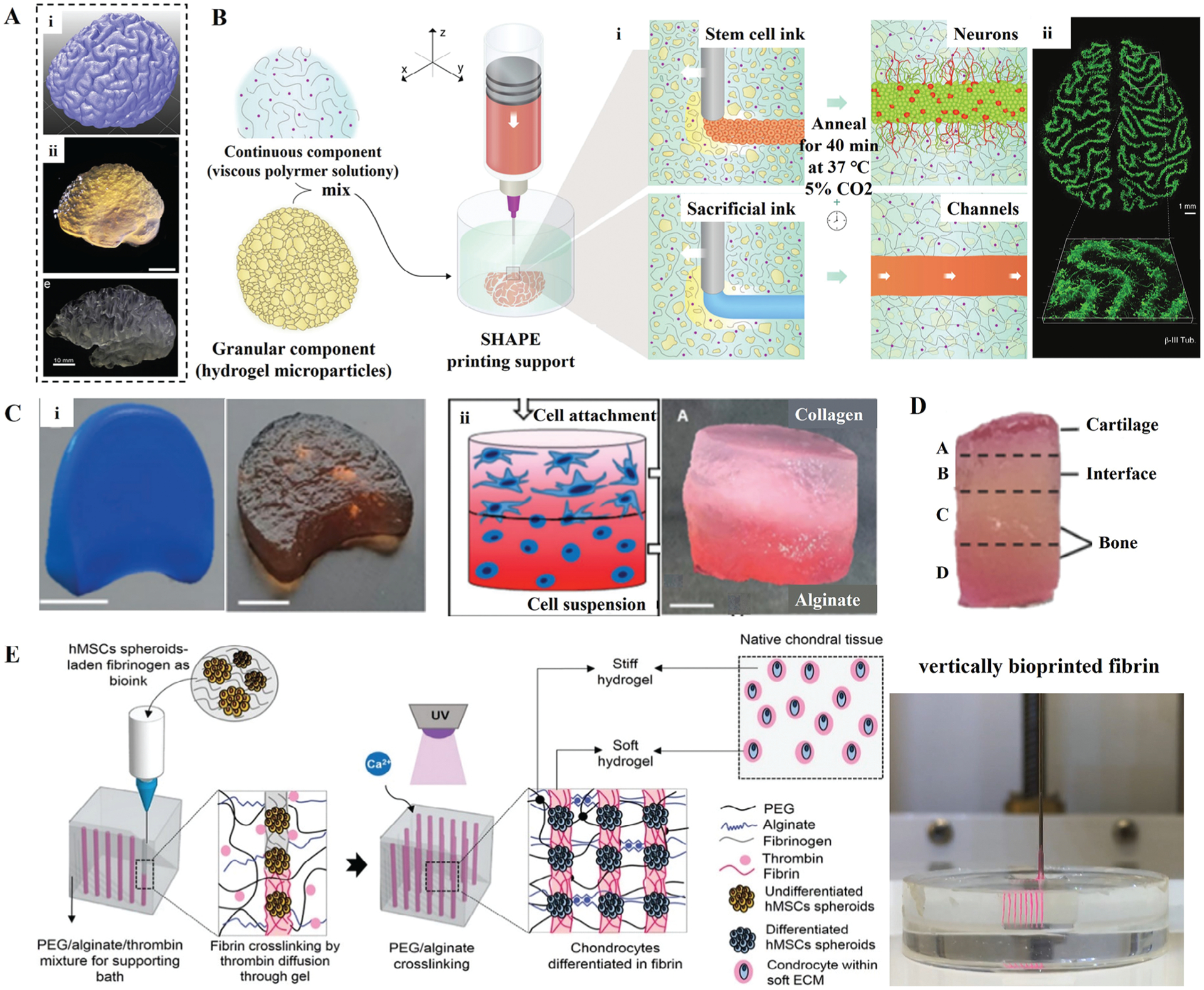
A) (i) 3D rendered human brain model from MRI data, (ii) a scaled down 3D printed brain model; Adapated with permission.^[24,117]^ Copyright 2015, American Association for the Advancement of Science and Copyright 2020, Elsevier. B) Embedded bioprinting in self-healing annealable composites for precise patterning of human neural constructs: (i) Graphical illustration of the bioprinting process in the SHAPE support bath. (ii) Fluorescence images of neurons in a 3D printed construct resembling brain meanders with a 3D close-up view of a selected segment; Adapated with permission.^[41]^ Copyright 2022, Wiley-VCH. C) Fabrication of (i) an intervertebral disc and (ii) multilayer gradient scaffolds using suspended layer additive manufacturing (SLAM). D) Bioprinted anisotropic osteochondral plugs; Adapated with permission.^[119]^ Copyright 2017, Wiley-VCH. E) 3D bioprinted cartilage-like constructs: schematic of the 3D bioprinting approach for the engineering of articular cartilage; Adapated with permission.^[109]^ Copyright 2019, Wiley-VCH.

Due to the remarkable capability of embedded bioprinting in generating complex structures, it has also been used to reconstitute other functional tissues. The profile of the human brain has been replicated, where the constructs showed complex external architectures that correspond to the white matter folds of the cerebral cortex (Figure 7A).^[24,117]^ Furthermore, the precise reconstruction of neural tissues with 3D geometry has become possible with the advance of embedded bioprinting. Kajtez et al.^[41]^ proposed a strategy, where a bioink loaded with human neural stem cells (hNSCs) were bioprinted in a self-healing particulate extracellular matrix (SHAPE) (Figure 7B). The SHAPE was composed of alginate particulate hydrogel and extra-cellular matrix (ECM) based solution (collagen, laminin, HA, and fibronectin), which provided both physical support for bioprinted constructs and biocompatible microenvironment for cellular growth. In particular, SHAPE was conducive to omnidirectional axonal elongation, promoting the development of neuronal constructs with delicate neuronal morphology and mature functional activity. Furthermore, this method was not limited to bioprinting simple shape structures but could be extended to structures with arbitrary designs, such as zigzag folds similar to brain rotation (Figure 7B).

Embedded bioprinting has also been applied to tissue models without built-in channels. For example, Jessica et al.^[47]^ created structures replicating bone and cartilage regions by bioprinting collagen, alginate, and gellan into agarose particles. Intervertebral discs and multilayer gradient scaffolds were also constructed (Figure 7C). Alioglu et al.^[118]^ reported a Matryoshka-inspired intra-embedded bioprinting method to develop nested bioprinted models by demonstrating embedded bioprinting inside pre-existing embedded bioprinted structures. Moxon et al.^[119]^ bioprinted anisotropic osteochondral implants with distinct mechanical properties (Figure 7D). The top layer was bioprinted with chondrocyte-laden gellan gum bioink, while the bottom layer was bioprinted with osteoblast-laden gellan gum bioink embedded with nanocrystalline hydroxyapatite. In addition, Melo et al.^[109]^ reported a cartilage-like tissue by bioprinting hMSC spheroid-laden fibrin in a stiff and tough biomaterial (i.e., PEG-alginate) (Figure 7E). Dual cross-linking of PEG-alginate resulted in interpenetrating polymer network (IPN) hydrogels, indicating great mechanical properties, resilience, and elasticity. The high diffusive capacity promoted the enzymatic cross–linking of fibrin and allowed nutrient diffusion throughout the stiff PEG-alginate hydrogel. Thus, the bioprinted cartilage-like tissue maintained a chondrogenic phenotype and mechanical properties similar to the native cartilage.

In summary, during the development of tissues and organs using embedding bioprinting, material systems, and process parameters should be customized accordingly. First, it is essential to select the proper materials to improve the bioprinting resolution and shape fidelity. The ideal support bath is expected to have controlled rheological properties and be easily removed, facilitating the bioprinting and extraction of complex 3D structures. Also, it is important to ensure the proper interactions between the bioink and support bath, which depends on the cross–linking mechanism. In addition, clear and intact constructs could be fabricated by regulating the printing parameters. The printing speed and bioink flow rate are crucial for obtaining high printing resolution and cellular viability simultaneously. Through manipulating the abovementioned factors, the accuracy and stability of bioprinted constructs are anticipated to be improved, which is beneficial for the regeneration of functional tissues and organs.

## 5. Post-Printing Treatment Methods

The last but equally important step in the process of embedded bioprinting is to extract the sacrificial material after bioprinting without impairing the bioprinted structure and its quality. There are several methods to extract the sacrificial material and it depends upon the material used for bioprinting and material to be sacrificed. Here, post-treatment methods are categorized into two sections that follow the removal of sacrificial support bath and ink.

### 5.1. Post-Treatment of Support Baths

The first post-processing strategy is to obtain the structure by sacrificing the support bath after bioprinting. The accuracy of the printed structure should ideally not be harmed during or after the removal of the support bath. One of the most prevalent methods is the direct wash of bioprinted constructs with an aqueous solution (e.g., Dulbecco’s phosphate-buffered saline or deionized water) ,^[47]^ which is typically employed in the particle-based support bath. For example, the Carbopol support bath can be removed by washing the bioprinted constructs with agitation in water.^[26,28]^ Although this mechanical method is simple and convenient, it may destroy the bioprinted structure and impair its mechanical strength. In addition, temperature-induced extraction is also a popular post-processing method. For example, gelatin particles support the embedded bioprinting of soft materials like collagen since gelatin is a thermally reversible hydrogel. It forms a stable gel at ambient temperature and melts at 37 °C, making it simple to remove. This strategy has been applied to successfully fabricate collagen-based human heart models.^[18]^ Furthermore, because of its hydrophilicity, polyethylene glycol (PEG400) is used to decrease the viscosity of the support bath. To get rid of the support bath containing H-HPMC and Pluronic, PEG400 could interpose between macromolecular backbones to reduce the material viscosity, thus achieving the purpose of dilution and removal of the support bath.^[32,37]^ However, it is equally essential to make sure that the additives do not affect the rheological properties of the support bath except for the effect of dilution. Nevertheless, it is still challenging to efficiently develop a stable interconnected complex 3D printed network in the tens of micrometer range, with one of the reasons being the difficult process of removing sacrificial support bath from micro pores of the 3D printed structure without damaging the microstructure itself. It has been reported that the mass of structures printed in a support bath was higher than that of the extruded bioink, although the printed structures were visually clean on the macroscopic scale, suggesting that some support material remained during the structure extraction.^[52]^ Zeng et al.^[120]^ investigated the residues of support materials from a microscopic perspective, where the residual support materials on fibrin fibers printed in different granular baths were compared, including gellan gum (7.5 μg mm^−2^), gelatin (0.3 μg mm^−2^) and polyvinyl alcohol baths (28–70 μg mm^−2^). Quantitative results indicated that the material residue or the space created by the removal of gel particles induced changes in surface morphology and mechanical properties of printed structures. Therefore, more flexible support bath materials are needed that allow 3D printing of < 10 μm fibers and in the meantime are more facile to remove afterward. Therefore, more flexible support bath materials are needed allowing 3D printing of < 10 μm fibers and simultaneously are more facile to remove afterward.

### 5.2. Post-Treatment of Sacrificial Inks

The second post-processing strategy is to obtain perfusable channels by the removal of the extruded ink, while the support bath, being part of the final structure, provides a biologically friendly environment for cells.^[28]^ Ideal sacrificial inks can have a mild and controllable gel-liquid transition property for removal after bioprinting, such as gelatin^[20,23]^ and Pluronic.^[22,77]^ For instance, sacrificial gelatin ink was extruded into living matrices composed of OBBs in the SWIFT process. After extrusion, a network of vascular channels was accomplished by melting and removing gelatin at 37 °C.^[23]^ Pluronic, a thermosensitive material liquefied at 4 °C and solidified at room temperature, has also been used as a sacrificial ink to create perfusable channels in 3D constructs.^[77]^ For this post-treatment method, it is desirable to have a low diffusion coefficient between the ink and the support bath to minimize unwanted variations in channel size. Ink diffusion in the support bath may result in the generation of undesirable tiny features while washing and removing the sacrificial ink, thus impairing the efficiency of this method.^[16]^ The removal of the sacrificial ink can also be realized by dilution, which is triggered through the interaction between chemical bonds. Song et al.^[113]^ used soluble beta-cyclodextrin (*β*-CD), which disrupted guest-host bonds, to remove the sacrificial ink, leaving behind a well-defined microchannel within the hydrogel.^[28,113]^ Properties of bioprinted structures vary when different post-processing techniques are chosen, which must be cell-friendly with no adverse effects on the bioprinted structures. Fulfilling all requirements for the post-treatment of sacrificial inks is a challenging process with an intricate series of steps. It is difficult but crucial to efficiently remove the sacrificial ink from long and thin vessels having branches as its toxic remnants can inhibit the biological functions of seeded cells and limit the vascularization of tissues. Research into advanced post-treatment methods of sacrificial inks is needed that can overcome the current challenges. In addition to alternative post-treatment methods, novel techniques of making perfusable channels would also be promising to explore.

## 6. Discussions and Outlook

Herein, we have discussed the process of embedded bioprinting and meticulously discussed the individual and combined influence of the associated parameters, such as support bath and bioink material rheology, printing parameters, cross–linking mechanisms, and post-processing strategies. Among these, the material’s rheological characteristics and printing parameters predominantly influence the embedded printing process more than the other parameters.^[29,30,33,34]^ The rheological properties of the support bath material are mainly characterized by its shear thinning and thixotropy/self-healing nature, yield-stress, modulus, and viscosity. Especially for a particle-based support bath, the distribution of particle size affects the rheological properties and subsequently the printing quality. Both process and design parameters need to be compatible with the specified bioinks and bioprinting conditions. Moreover, by applying a suitable cross–linking methodology and post-printing treatment method, it is possible to achieve the required print fidelity. In addition to considering the impact of these aspects on the fidelity and resolution of bioprinted structures, biocompatibility needs to be always taken into account to ensure the growth, maturation, and differentiation of bioprinted cells.

Despite numerous research findings reported in recent years in terms of process development and tissue engineering applications, certain issues still require attention. The type of a support bath has been a major concern since the rheological properties of the support bath significantly affect the printing accuracy, stability, and fidelity. An ideal support bath material should exhibit excellent tunability, stability, and biocompatibility to support the bioprinting of multiple bioinks for a wide range of applications.^[121,122]^ For example, the H-HPMC support bath composed of hydrophobically modified hydroxypropylmethyl-cellulose and Pluronic allows for five common cross–linking methods (i.e., photo, enzymatic, ionic, pH, and thermal cross–linking ), enabling multi-material bioprinting with multiple cross–linking strategies.^[37]^ Also, the SHAPE support bath enabled the high fidelity of bioprinted structures with fine features, which were bioprinted using three types of bioinks comprising different stem cells (hNSCs from ventral mesencephalic and hNSCs from forebrain region), simulating the interactions between different cell types in the brain.^[41]^ In another study, a cellulose nanocrystal (CNC)-based bath supported the bioprinting of multiple cell types and perfusable channels, showing the potential for manufacturing microfluidic perfusion bioreactors that incorporated different cell types.^[95]^ The development of these support bath materials creates effective platforms for multi-material embedded bioprinting, laying the groundwork and opening up prospects for heterogeneous tissue and organ fabrication.

In addition, existing support bath materials are usually designed for bioprinting of small constructs (i.e., < 1 cm^3^), while those for large-scale and batch bioprinting have been hardly reported. Large-scale tissue bioprinting takes more time to execute, raising the challenge of maintaining the viability of bioprinted cells. For example, cellular viability decreased from 80 to 25 and 6% as the total bioprinting time increased from 30 to 45 and 60 min when it was cured by an Irgacure 2959 crosslinker.^[123]^ In this regard, more efforts are needed to design the production process for making large batches of support materials that are convenient, cell-friendly, and cost-efficient. In addition to increasing the production efficiency of support bath materials, researchers should also focus on developing reusable support bath materials to reduce the cost of embedded bioprinting for scalable constructs. For instance, it has been shown that HA-based support materials are advantageous in being relatively easy to prepare and store. Further, the same support bath material can be reused in the bioprinting process three times without eliciting significant differences among the three prints made in the same support bath.^[40]^

Another critical factor is the selection of the right nozzle, which also impacts the bioprinting results. The bioprinting process may be interrupted because of nozzle blockage due to a highly viscous bioink or bending due to the resistance from the support material, which is catastrophic for the bioprinting efficiency and quality. Hence, nozzle optimization, in terms of shape and material to improve mechanical stability, is necessary. Nozzles of steel alloy reinforced with stainless steel have been used to reduce the deflection during motion.^[18]^ Experimental results showed that the customized nozzles reinforced with an outer tube of alloy steel and a needle collar were used to successfully bioprint an adult human heart scaffold in a large gelatin support bath, accounting for the increased needle drag during bioprinting.^[18]^ In addition, a curved nozzle was used to prevent the flow disturbance of bioinks, ensuring print quality.^[63]^ A novel nozzle design based on a telescopic structure was also developed to satisfy embedded bioprinting. To maintain a robust needle structure and print quality, metal tubes with different diameters were combined with telescopic structures to support the inner nozzle with a small diameter.^[124]^

Regarding the degree of freedom, although most bioprinters are equipped with three axes (*x*, *y*, and *z* axes),^[125]^ advancing the bioprinters with a robotic arm has also been reported. A robotic arm, owing to its six axes (three orthogonal and three rotational axes), provides six degrees of freedom that offer more flexibility than conventional bioprinters, thus expanding the capability of embedded bioprinting.^[126,127]^ At present, commercial bioprinters (e.g., BioAssemblyBot) have shown the potential of irregular-shaped printing and intraoperative bioprinting.^[128]^

In the bioprinting process, different but highly relevant parameters (e.g., flow rate, printing speed, and nozzle size) have to be optimized based on numerical simulation or experiments.^[129]^ In particular, for the production of large constructs with simple features, there is a trade-off among the parameters, including printing speed, feature size, and printing time.^[80]^ Therefore, it is of great interest to develop a predictive algorithm targeting the optimization of embedded bioprinting parameters, such as hierarchical machine learning (HML).^[79]^ Experimental results revealed that the bioprinting parameters optimized by HML improved the printing speed by 2.5 times without sacrificing the print fidelity compared to trial and error generated parameters. In a recent study, Joel et al.^[130]^ investigated the FRESH method to bioprint sodium alginate using a self-designed convolutional neural network (CNN) to classify the quality of the bioprinted fibers. This deep learning model with convolutional neural networks could perform process monitoring and thus identify the quality of bioprinted structures. In addition to the predictive models for judging the final printing patterns, it is also of interest to involve bioprinting process monitoring and visualization to further assess or even quantify the printing fidelity.^[18,26,31]^

As to the post-bioprinting extraction, it is important to completely remove the bioprinted structures without any remnants of support bath materials, especially for structures with small features. Therefore, it is essential to develop solid-liquid reversible conversion materials (with the assistance of temperature, pH, or enzyme reversibility) that facilitate the post-processing steps and reduce the impact on bioprinted structures. The swelling and shrinkage during post-processing also affect the printing resolution, which is often overlooked. For example, a post-processing shrinkage phenomenon has been utilized to improve the printing resolution.^[40,131]^ Hydrogel constructs bioprinted using anionic bioinks (such as GelMA and alginate) are immersed in a polycationic chitosan solution, occurring charge complexation and subsequent expulsion of water from the gels. Although a reduction in dimensions was obtained without changing bioprinting parameters or material properties, it may still be challenging to accurately control the different shrinking rates and widen the adaptability for various material systems. Overall, these above-mentioned limitations make it clear that improving post-processing strategies to ensure print quality and resolution will be necessary to advance embedded bioprinting in the future.

## 7. Conclusions

Embedded bioprinting is an emerging bioprinting technology that has exhibited immense potential to fabricate multi-material complex structural organization within a support bath. It has significantly widened the materials range for bioprinting by involving low viscosity or soft materials as well as direct bioprinting of individual cells and spheroids thus opening up a plethora of possibilities in developing viable scalable constructs. Crucial factors that affect the print fidelity, resolution, and stability involve the rheological properties of the support bath and bioink materials, process parameters, cross–linking mechanism, and post-treatment methods, which are comprehensively discussed in the presented work. It will provide systematic references and directions to design a complete embedded bioprinting platform including selection of bioinks, support materials, and printing parameters that are ideal for the targeted biomedical applications. It is believed that new advances in embedded bioprinting will provide promising platform technologies for the biofabrication of functional solid organs in the future.

## Figures and Tables

**Table 1.** List of μP-based support bath materials along with respective bioinks used for embedded bioprinting.

Particle-based support bath materials		
Hydrogel μP	Bioink	References
Gelatin	Alginate, modified alginate	[18,19,24,47,132–143]
	Xanthan Gum	[43]
	Decellularized extracellular matrix (dECM)	[144,145]
	Embryoid body matrix	[23]
	Gelatin methacrylate (GelMA)/ poly(3,4-ethylenedioxythiophene):poly(styrenesulfonate)	[146]
	Collagen	[18,24,47,56,140–142,144,147–153]
	Fibrin, Fibrinogen	[24,56,148,154]
	Hyaluronic acid (HA), functionalized/modified HA	[18,149,154–158]
	Cells, spheroids	[18,155]
	Locust bean gum / Paracetamol Locust bean gum / Ibuprofen	[159]
	i-carrageenan	[47]
	Gellan gum	[47]
	Cellulose nanofibrils / Carbon nanotubes / Alginate	[160]
	Polyethylene glycol (PEG), functionalized PEG	[114,148,155,161,162]
	Laminin	[150]
	Gallium-Indium / Alginate, Polyester, Silk, Stainless steel	[140]
	Calcium phosphate	[163]
	Carbopol	[142]
	Pectin	[157]
	Gelatin or GelMA-based	[36,148,155]
	Silk	[164]
	Pluronic	[77,143]
	Methylcellulose	[77]
	Polyvinyl alcohol (PVA)	[77]
	Poly(ethylene glycol) diacrylate (PEGDA)	[143]
Alginate	Xanthan gum	[43,165]
	Cells, spheroids	[41,166,167]
	Gelatin or GelMA-based	[41,110,165]
	Silicone, Poly(dimethylsiloxane) (PDMS)	[168,169]
	Alginate	[41]
Agarose	Alginate	[45,47,95]
	Gelatin or GelMA-based	[45,74,95]
	Collagen	[45,47]
	Platelet lysate	[95]
	Thrombin, aldehyde-functionalized cellulose nanocrystals (CNC)	[170]
Polyacrylamide/PEDGA	Collagen	[171]
Polytetrafluoroethylene	Cellulose nanofiber / Mannitol / Bacteria	[172]
GelMA	Pluronic F-127	[173]
Agar	Alginate, Alginate-RGD, Alginate-RGD / Soy protein isolate Alginate-RGD / Pea protein isolate	[174]
Aldehyde functionalized poly(ethylene glycol methacrylate) /Gelatin	hydrazide functionalized poly(ethylene glycol methacrylate)	[36]

Gellan	Collagen, Fibrinogen	[56,136]

**Table 2.** List of continuous-phase support bath materials along with respective bioinks used for embedded bioprinting.

Continuous phase support bath materials		
Support bath material	Bioink	References
Pluronic-based	Pluronic, modified Pluronic	[22,61]
	Alginate	[32,37,62,175,176]
	Gelatin or GelMA -based	[32,37,103,155,176]
	Collagen	[32,37]
	Polyelectrolyte complex	[177]
	PDMS, Silicone	[178]
	*α*-calcium phosphate / Pluronic P123, *α*-calcium phosphate / hydroxypropyl methylcellulose	[179]
Silicone-based	Carbon conductive grease	[72]
	Photocrosslinkable PVA	[180]
	Water/carboxylic acid-functionalized silica nanoparticles	[181]
	PDMS, Silicone	[81,180,182,183]
	Xanthan gum	[65,184]
	Pluronic	[184]
Hyaluronic acid (HA)-based	HA, modified HA	[59,60,79,185]
	Spheroids	[186]
	GelMA	[79]
	Modified HA / PEDGA / Agarose μP	[133]
Carbopol	PVA	[180]
	PDMS, Silicone	[49,87,117,129,180,187–189]
	Alginate	[143,190–193]
	Gelatin or GelMA-based	[26,79,190,191,194]
	Urethane Plastic, Urethane Rubber	[195]
	Liquid metal	[196]
	PEG μP	[197]
	Collagen	[198]
	Dimethyl itaconate / Triethyl citrate / 1,8-octanediol / Poly(ethylene glycol) dimethy| ether	[48]
	Spheroids	[167]
	HA, modified HA	[79,199]
	Thermoplastics	[199]
	Calcium phosphate / Hydroxyapatite cement	[193]
	CNC	[200]
	Epoxy	[201]
	PEGDA	[143]
Agarose-based	Gellan	[47,202,203]
	Pluronic	[204,205]
	Alginate	[47,143,206]
	Collagen	[47]
	Gelatin-based	[79,205,207,208]
	HA, modified HA	[79]
	Dextran-Methacrylate (DexMA)	[205]
	PEGDA	[143]
Cellulose nanofiber	Petroleum jelly	[195]
Polyethylene glycol (PEG)-based	Silk	[105]
	PDMS	[209]
	Dextran / Poly-l-lysine	[114]
	Alginate, Alginate / Polyethylene glycol dimethacrylate, Collagen, Gelatin	[71]
Xanthan gum	Gelatin or GelMA-based	[210,211]
	dECM	[210]
	Alginate	[211]
	CNC	[211,212]
	HA, modified HA	[213]
	poly(lactic acid) (PLA) μP, Collagen / PLA μP/ Spheroids, Collagen / PLA μP/ Cells, Spheroids, Cells	[63]
Dextran / Polyethylene oxide (PEO) Dextran / Polyacrylamide / PEO Dextran / poly(acrylic acid) / PEO PVA	Dextran / Polyethylene oxide (PEO) Dextran / Polyacrylamide / PEO Dextran/poly (acrylic acid) / PEO PVA	[214]
Gellan-based	Gelatin / Gellan, Gelatin / Alginate, Alginate, PEGDA	[52]

**Table 3.** Comparison of nozzle size, printing speed, and resolution reported for embedded bioprinting.

Inner needle diameter (μm)	Printing speed [mm ^−1^ s]	Minimum fiber diameter [μm]	References
10–200	6	18	[22]
60, 200, 610	0.5–4	35	[59]
50, 200	0.025–3.7	100	[180]
400	20	140	[49]
150–3000	50	1000	[195]
100, 150, 200, 250, 330, 400	0.5–15	≈100	[27]
150–1000	0.1–10	≈75	[215]
60–410	10–100	250	[196]
180–610	2–200	300	[105]
210	8.33	600	[43]
410	160–400	500	[216]
160, 260, 1600	1.67–66.67	45	[181]
10–200	3–23	20	[18]
400, 500, 800	0.5, 1, 1.5	400	[58]
200, 250, 410	0.66–2.66	300	[145]
150100260	30	140	[204]
150	0.66–3.33	60	[191]
250	0.5–4	400	[23]
150	5–10	120	[146]
110–1550	8–18	200	[206]
10–1730	0.83–1.66	200	[214]
150	2,4,6,8	300	[135]
250	2.5	250	[203]
100–610	0.5–10	30	[217]
150	0.1–0.5	50	[171]
150	1–10	500	[172]
150	5–10	500	[110]
150200	0.1–2	202	[198]
200	10	200	[26]
0.610	15	≈700	[218]
150	1–20	120	[161]
27	5	34	[95]
200	1,2,3	200	[176]
330	1.5–6	250	[219]
150–410	8, 12	250	[220]
150–400	0.167–0.583	200	[221]
370480590680	1,3,6,12,20	100	[37]
250	2–5	255	[103]
200, 510	0.3–1	200	[41]
152–410	1–3	84	[63]
310–510	4–12	325	[74]
260	0.416–8.33	120	[177]
110160210	1.6–25	200	[114]
610, 840, 1540	0.75–3	150	[222]
60–680	2–20	50	[32]
410	2	230	[77]
410	0.75–4.5	50	[178]
150	2–10	182	[223]
100, 410	0.25–2	50	[65]
60	0.25–2	30	[184]
150–250	5–30	≈100	[170]

**Table 4.** Different cross–linking mechanisms along with their respective bioinks and support baths reported for embedded bioprinting.

Cross-linking mechanism	Bioink	Support bath
Photo	Alginate / GelMA,^[176]^ GelMA^[32,37,103]^ , Polyelectrolyte complex,^[177]^ poly(acrylamide) μP^[222]^	Pluronic-based
	Modified HA^[59,60]^	Hyaluronic acid-based
	Modified HA^[156,157]^ , GelMA/poly(3,4-ethylenedioxythiophene): poly(styrenesulfonate) ,^[146]^ GelMA, GelMA / collagen methacrylate,^[148]^ Poly(ethylene glycol) methacrylate^[162]^	Gelatin μP
	GelMA-based^[45,95,205,207,208]^ , PEGDA/Acrylamide^[143]^	Agarose
	Photocrosslinkable PVA,^[180]^ GelMA-based^[26,191,194]^ , Photocrosslinkable CNC,^[200]^ Photocroslinkable resin,^[221]^	Carbopol
	Photocrosslinkable silicone^[81,182,215,224,225]^ , Photocroslinkable resin,^[217]^ Xanthan Gum/ Pluronic^[184]^	Silicone-based or mineral oil-based
	Cells,^[166]^ Alginate/CNC/ GelMA,^[211]^ GelMA^[165,226]^ , PEGDA,^[41]^ Photocrosslinkable PVA^[212]^	Alginate-based
	N-isopropylacrylamide / Alginate, Acrylamide / Alginate^[58]^	Carboxymethyl cellulose
	Gelatin / Gellan, Gelatin / Alginate, Alginate, PEGDA^[52]^	Gellan, Laponite
	Cetylpyridinium chloride monohydrate / PEDGA^[220]^	Castor oil / Oleic Acid
	Alginate/ Gelatin/ GelMA, Laminin,^[223]^ *α*-calcium phosphate^[227]^	GelMA-based
	Polyethylene glycol dimethacrylate/Alginate^[71]^	Gelatin
Thermal	Collagen-based^[32,37]^	Pluronic-based
	Collagen-based^[18,24,144,149–152]^ , dECM,^[145]^ Chitosan/Gelatin^[36]^	Gelatin μP
	Collagen^[198]^	Carbopol
	Gellan / hydroxyapatite^[202]^	Agarose
	dECM-based^[210,228]^ , Collagen-based^[229]^	Alginate-based
	Alginate / Gelatin^[27,216]^	Laponite
	Alginate / Collagen^[45]^	Agarose μP
	Locust bean gum / Paracetamol Locust bean gum / Ibuprofen,^[159]^ Collagen-based^[147]^	Gelatin
	Collagen, Gellan gum, Alginate, i-carrageenan^[47]^	Agarose, Gelatin μP, Agarose μP
	Alginate / Carrot, Chocolate, Dough^[192]^	Carbopol / Gelatin μP
	Alginate, Collagen, Gallium−Indium / Alginate, Polyester, Silk, Stainless steel^[140]^	Gelatin μP, Alginate, Collagen
	Cells^[230]^	Collagen, Collagen / Matrigel
	PLA μP, Collagen / PLA μP/ Spheroids, Collagen / PLA μP/ Cells, Spheroids, Cells^[63]^	Alginate / Xanthan gum
	PEDGA, Acrylamide / Alginate, poly(acrylic acid) / PEDGA, Chitosan / PEDGA, Alginate^[143]^	Carbopol, Agarose, Gelatin μP, Fumed silica/mineral oil, Oleic acid / Stearic acid
Ionic	Alginate-based^[18,19,24,132,134,135,137–139,156,160,191]^ , GelMA / poly(3,4-ethylenedioxythiophene): poly(styrenesulfonate),^[146]^ Alginate^[136]^	Gelatin μP-based
	Alginate-based^[190,193]^	Carbopol
	Alginate-based^[27,99,216]^ , Gelatin / Gellan, Gelatin / Alginate, Alginate, PEGDA^[52]^	Laponite-based
	Alginate-based^[32,37,62,175,176]^	Pluronic-based
	dECM-based^[210,228]^ , Cells,^[166]^ Alginate/CNC / GelMA, GelMA,^[211]^ Alginate, Collagen, Gallium−Indium / Alginate, Polyester, Silk, Stainless steel,^[140]^ Alginate, Alginate / Polyethylene glycol dimethacrylate, Collagen, Gelatin, PEG^[32]^	Alginate-based
	Alginate-based^[58]^	Carboxymethyl cellulose
	Alginate-based^[45,174]^	Agarose μP
	Gellan / hydroxyapatite,^[202]^ Gellan, Laponite / Gellan^[203]^	Agarose
	Alginate^[206]^	Agarose / Gelatin, Gelatin, GelMA
	Xanthan Gum^[43]^	Alginate μP
	Collagen, Gellan gum, Alginate, i-carrageenan^[47]^	Agarose, Gelatin μP, Agarose μP
	Cellulose nanofiber / Mannitol / Bacteria^[172]^	Polytetrafluoroethylene μP
	Alginate, CNC, Gelatin, GelMA, Platelet lysate^[95]^	CNC, CNC / Gelatin, CNC/Alginate, CNC/GelMA, Agarose μP
	Pollen / Alginate, Alginate, PDMS^[219]^	Pollen, Water
	Gelatin, Alginate, PEGDA, Cells^[41]^	Alginate μP/ Collagen
	Alginate/ Gelatin/ GelMA, Laminin^[223]^	GelMA
	PEDGA, Acrylamide / Alginate, poly(acrylic acid) / PEDGA, Chitosan / PEDGA, Alginate^[143]^	Carbopol, Agarose, Gelatin μP, Fumed silica/mineral oil, Oleic acid / Stearic acid
Enzymatic	Fibrin^[18,24]^	Gelatin μP
	Alginate^[206]^	Agarose / Gelatin, Gelatin
	Gelatin / Gellan, Gelatin / Alginate^[52]^	Gellan, Laponite
	Alginate, CNC, Gelatin, GelMA, Platelet lysate^[95]^	CNC, CNC/Gelatin, CNC/Alginate, CNC/GelMA, Agarose μP
	Gelatin^[41]^	Alginate μP/ Collagen
	Methylcellulose, Pluronic F-127, PVA^[77]^	Gelatin μP/ Gelatin
Chemical	Carbon conductive grease,^[72]^ Silicone-based^[27,81,181–183,218]^ , Pluronic-based,^[73]^ Xanthan gum^[65]^	Silicone-based or Mineral oil-based
	Silicone-based^[49,87,129,187–189]^, Epoxy^[201]^	Carbopol
	Pluronic-based,^[61]^ Silicone-based^[178,209]^ , *α*-calcium phosphate / Pluronic P123, *α*-calcium phosphate / hydroxypropyl methylcellulose^[179]^	Pluronic-based
	Xanthan Gum,^[43]^ PEG,^[161]^ Fibrinogen, HA, Fibrinogen / HA,^[154]^ Calcium phosphate,^[163]^ Silk fibroin,^[164]^ Alginate,^[136]^ Collagen, Fibrinogen^[56]^	Gelatin μP-based
	Carbohydrazide-Modified Gelatin^[110]^	Oxidized alginate/Gelatin μP
	Silicone-based^[168,169]^	Alginate μP
	Silicone-based^[219]^	Pollen, Water
	Modified HA^[213]^	Xanthan Gum
	Silicone^[231]^	Laponite
	Hydrazide functionalized poly (ethylene glycol methacrylate)^[232]^	Aldehyde functionalized poly(ethylene glycol methacrylate) / Gelatin μP
	Dextran / Poly-l-lysine^[114]^	Oxidized bacterial cellulose / PEG
	Thrombin, aldehyde-functionalized CNC^[170]^	Agarose μP
